# Defining the *Hoxb8* cell lineage during murine definitive hematopoiesis

**DOI:** 10.1242/dev.200200

**Published:** 2022-04-22

**Authors:** Donn A. Van Deren, Shrutokirti De, Ben Xu, Kayla M. Eschenbacher, Shuhua Zhang, Mario R. Capecchi

**Affiliations:** 1 Department of Human Genetics, University of Utah School of Medicine, Salt Lake City, UT 84112, USA; 2 Interdepartmental Program in Neuroscience, University of Utah School of Medicine, Salt Lake City, UT 84112, USA

**Keywords:** *Hoxb8* lineage, Hematopoiesis, Obsessive-compulsive spectrum disorders, Anxiety

## Abstract

Previously, we have demonstrated that a subpopulation of microglia, known as *Hoxb8* microglia, is derived from the *Hoxb8* lineage during the second wave (E8.5) of yolk sac hematopoiesis, whereas canonical non-*Hoxb8* microglia arise from the first wave (E7.5). *Hoxb8* microglia have an ontogeny distinct from non-*Hoxb8* microglia. Dysfunctional *Hoxb8* microglia cause the acquisition of chronic anxiety and an obsessive-compulsive spectrum-like behavior, trichotillomania, in mice. The nature and fate of the progenitors generated during E8.5 yolk sac hematopoiesis have been controversial. Herein, we use the *Hoxb8* cell lineage reporter to define the ontogeny of hematopoietic cells arising during the definitive waves of hematopoiesis initiated in the E8.5 yolk sac and aorta-gonad-mesonephros (AGM) region. Our murine cell lineage analysis shows that the *Hoxb8* cell lineage reporter robustly marks erythromyeloid progenitors, hematopoietic stem cells and their progeny, particularly monocytes. *Hoxb8* progenitors and microglia require *Myb* function, a hallmark transcription factor for definitive hematopoiesis, for propagation and maturation. During adulthood, all immune lineages and, interestingly, resident macrophages in only hematopoietic/lymphoid tissues are derived from *Hoxb8* precursors. These results illustrate that the *Hoxb8* lineage exclusively mirrors murine definitive hematopoiesis.

## Abstract (precis)

**Summary:** The *Hoxb8* cell lineage reporter provides new insights into the cellular ontogeny of the murine immune system.

## INTRODUCTION

There are at least three successive waves of hematopoiesis in the developing mouse embryo (Ginhoux and Guilliams, 2016; Godin et al., 1995; Hoeffel and Ginhoux, 2015). The activity of these hematopoietic waves temporally overlaps. The first wave initiates in the blood islands of the yolk sac. Starting at embryonic day (E) 7.5, this primitive wave generates erythromyeloid progenitors (EMPs), which includes hematopoietic progenitors for canonical non-*Hoxb8* microglia that directly migrate to and colonize the developing brain at E9.5 (Ginhoux et al., 2010). The second wave initiates in the hemogenic endothelium of the yolk sac. This definitive wave generates a second population of EMPs at E8.5, including the *Hoxb8* microglia progenitors that proceed through the aorta-gonad-mesonephros (AGM) region and fetal liver before they colonize the developing brain starting at E12.5 (De et al., 2018). Together, non-*Hoxb8* microglia and *Hoxb8* microglia serve as the immune cells of the brain. It is this latter subpopulation of microglia that, when dysfunctional, results in the acquisition of chronic anxiety and obsessive-compulsive spectrum-like behavior (i.e. either genetic disruption of *Hoxb8* or specific ablation of this microglial subpopulation in mice results in chronic anxiety and trichotillomania-like pathology) (Nagarajan et al., 2018; Trankner et al., 2019). A third, definitive wave initiates in the hemogenic endothelium of the para-aortic splanchnopleura (P-Sp), which then becomes the AGM region and is presumed to generate pre-hematopoietic stem cells (pre-HSCs) starting at E8.5 (Cumano and Godin, 2007).

The ontogeny of tissue-resident macrophages, including microglia, is under debate. For example, although it is believed that yolk sac EMPs generate microglia through yolk sac macrophage intermediates, it is still being debated whether these yolk sac EMPs originate from the first wave, second wave or both waves of yolk sac hematopoiesis. Recently, several models have been put forward that propose different origins and ontogenies of tissue-resident macrophages and microglia. The first model, proposed by Hoeffel et al., states that two waves of EMPs exist in the yolk sac during yolk sac hematopoiesis. The first wave of EMPs (E7.5) gives rise to microglia via yolk sac macrophage intermediates, whereas the second wave (E8.5) gives rise to tissue-resident macrophages via fetal monocytes. EMPs born in the E8.5 yolk sac do not require colony-stimulating factor 1 receptor (*Csf1r*) expression for survival but do require *Myb* expression (Hoeffel et al., 2015; Kierdorf et al., 2013). *Csf1r* is essential for regulating the development of myeloid cells (Stanley and Chitu, 2014) and is necessary for the viability of microglia (Elmore et al., 2014). *Myb* is required to establish hematopoiesis in the fetal liver and the renewal of adult HSCs (Mucenski et al., 1991; Schulz et al., 2012; Sumner et al., 2000). Most E8.5 EMPs vacate the yolk sac and seed the primordial fetal liver by E9.5, where they differentiate into fetal liver monocytes and are proposed, in this model, to be the precursors of most tissue-resident macrophages (Ginhoux and Guilliams, 2016; Hoeffel et al., 2015).

The second model, proposed by Gomez Perdiguero et al., argues that all EMPs are born in the yolk sac at E8.5 and differentiate into yolk sac macrophages that, in turn, populate all tissues of the developing embryo, including the brain, to become tissue-resident macrophages and microglia, respectively (Gomez Perdiguero et al., 2015; Kierdorf et al., 2015; Perdiguero and Geissmann, 2016). In this model, fetal monocytes in the fetal liver, whether derived from yolk sac EMPs or AGM-derived fetal-HSCs (fHSC), minimally contribute to tissue-resident macrophage populations, including microglia. Interestingly, *Myb* is expressed in E8.5 EMPs and has been suggested to be required to differentiate erythroid, but not myeloid, progenitor cells (Perdiguero and Geissmann, 2016). In the fetal livers of *Myb*-null embryos, there is an absence of fetal liver monocytes, but fetal macrophages remain present (Schulz et al., 2012), suggesting that fetal macrophages may arise from a *Myb*-independent lineage originating in the yolk sac (i.e. E8.5 EMPs). These results may not exclude the possibility that fetal liver monocytes can give rise to fetal macrophages (Hoeffel et al., 2015; Sheng et al., 2015).

Another hematopoietic site within the embryo proper has been suggested to generate pre-HSCs at E8.5 in the P-Sp region (third wave of hematopoiesis). The third model, proposed by Sheng et al., states that all tissue-resident macrophages arise from fHSCs. Nascent pre-HSCs that mature into fHSCs begin seeding the primordial fetal liver by E10.5. Like E8.5 yolk sac EMPs, fHSCs also contribute to the generation of fetal liver monocytes in the developing liver (Ginhoux and Guilliams, 2016; Hoeffel et al., 2015; Sheng et al., 2015). By E14.5, fetal liver monocytes exit the fetal liver to populate all tissues of the developing embryo except the brain (Hoeffel et al., 2015).

The models proposed above agree that EMPs generate microglia. One debate centers around whether there are two distinct waves or one continuous wave of EMPs in the yolk sac. The ontogeny of tissue-resident macrophages, whether these cells differentiate from yolk sac macrophages or fetal liver monocytes, also remains unclear. Adding to this complexity, the precise origin of fetal liver monocytes is uncertain because of the contribution from AGM-derived fHSCs (Sheng et al., 2015) and yolk sac-derived EMPs (Hoeffel et al., 2015).

Are there additional Cre drivers that could help clarify the proposed models of the ontogeny of tissue-resident macrophages? The *Hoxb8-IRES-Cre* driver has been shown to label immune cell populations in various tissues, including blood, bone marrow and brain (Chen et al., 2010; De et al., 2018; Trankner et al., 2019). However, the extent to which this Cre driver labels immune cell populations, including tissue-resident macrophages and their hematopoietic progenitors, has not been described.

In this comprehensive study, we define the ontogeny of the immune cells of the *Hoxb8* lineage using the *Hoxb8-*tdTomato lineage reporter mouse described by De et al. (2018)*.* During embryonic development, the *Hoxb8* cell lineage marker exclusively marks the definitive hematopoietic waves of the yolk sac (E8.5) and AGM (E10.5), both of which are fundamentally distinct from the primitive hematopoietic wave of the yolk sac (E7.5). In support of the Hoeffel model, we show that fetal monocytes are exclusively derived from the *Hoxb8* lineage, whereas yolk sac macrophages are not, implying that *Hoxb8* fetal monocytes contribute to the generation of tissue-resident macrophages. We show that the *Hoxb8* cell lineage marker labels adult tissue-resident macrophages preferentially located in hematopoietic/lymphoid tissues. In contrast to the *Myb* independence of canonical non-*Hoxb8* microglia, we further describe a requirement of *Myb* for the propagation and maturation of *Hoxb8* hematopoietic progenitors and their intermediates, including *Hoxb8* microglia, during embryonic development. We propose that the *Hoxb8* cell lineage reporter robustly defines the ontogeny of definitive hematopoiesis.

## RESULTS

### *Hoxb8* lineage CD16/32^+^ EMPs become the dominant EMP population in the fetal liver

**Fig. 1.**
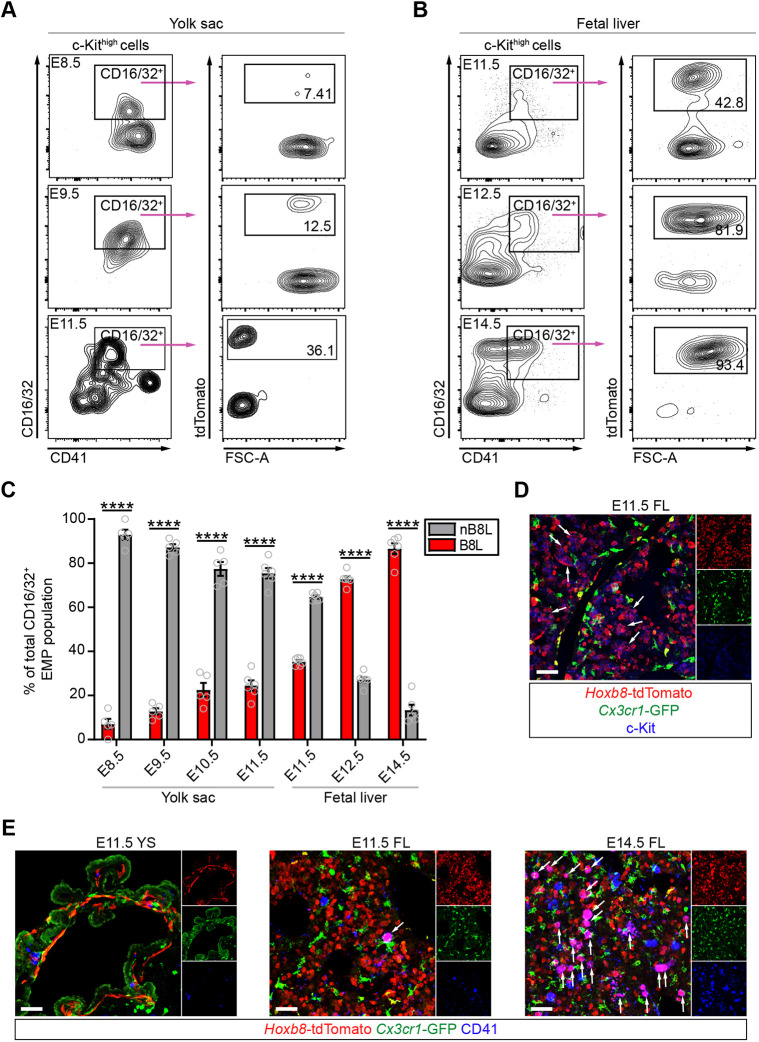
***Hoxb8* lineage CD16/32^+^ EMPs become the dominant EMP population in the fetal liver.** (A,B) Representative flow cytometry plots of EMPs (Kit^high^ CD16/32^+^ CD41^+^) gated for tdTomato from (A) yolk sac (E8.5, E9.5 and E11.5) and (B) fetal liver (E11.5, E12.5 and E14.5). (C) Percentage of *Hoxb8* (B8L) and non-*Hoxb8* (nB8L) CD16/32^+^ EMPs during development in the yolk sac (YS) and fetal liver (FL). E8.5 YS, E9.5 YS and E10.5 YS: *n*=5 pooled biological replicates. E11.5 YS, E11.5 FL, E12.5 FL and E14.5 FL: *n*=6 biological replicates. Two-way ANOVA with post-hoc analysis comparing nB8L cells with B8L cells across all time points examined: ****P<0.0001. Gray circles are individual data points. Data are mean±s.e.m. Data are from two or three independent experiments per time point. (D,E) Representative images of YS and FL sections from E11.5 and E14.5 embryos marked with (D) *Hoxb8*-tdTomato, *Cx3cr1*-GFP and Kit, and (E) *Hoxb8*-tdTomato, *Cx3cr1*-GFP and CD41 antibodies. *n*=3 biological replicates per time point. Scale bars: 50 µm. White arrows indicate tdTomato^+^ GFP^−^ cells that are (D) Kit^+^ or (E) CD41^+^.

Previously, we identified the earliest developmental time point at which *Hoxb8* is expressed during embryonic hematopoiesis. We detected *Hoxb8*-tdTomato^+^ cells in the CD41^+^ Kit^+^ hematopoietic lineage in the yolk sac as early as E8.5 (De et al., 2018), coincident with the emergence of E8.5 definitive EMPs (CD41^+^ Kit^+^ CD16/32^+^) (McGrath et al., 2015). CD16/32, proposed by McGrath et al., distinguishes between primitive E7.5 yolk sac EMPs (CD16/32^−^ CD41^+^ Kit^+^) and definitive E8.5 yolk sac EMPs (CD16/32^+^ CD41^+^ Kit^+^). CD16/32^+^ EMPs also lack Sca-1 expression, which renders them distinct from HSCs. Combining these markers with the *Hoxb8*-tdTomato lineage reporter (*Hoxb8^IRES-Cre/+^; Rosa26^CAG-LSL-tdTomato/+^*), we were able to follow the consumption and expansion of EMP subpopulations. The amount of tdTomato^+^ EMPs were similar in the E8.5 yolk sac (CD16/32^−^, 3.42%; CD16/32^+^, 7.07%) and E8.5 embryo proper (CD16/32^−^, 0.42%; CD16/32^+^, 0.00%); however, the percentage of *Hoxb8* CD16/32^+^ EMPs significantly increased 4.1-fold compared with *Hoxb8* CD16/32^−^ EMPs in the E9.5 yolk sac (CD16/32^−^, 3.12%; CD16/32^+^, 12.76) (Fig. S1A,B). E8.5 CD16/32^+^ EMPs, which include a subset that is tdTomato^+^ (7.07%) (Fig. 1A,C), have been shown to exit the yolk sac to seed and proliferate in the fetal liver by E9.5 (Hoeffel et al., 2015; McGrath et al., 2015). At E11.5, the fetal liver already contains Kit^+^ cells that are primarily tdTomato^+^ (De et al., 2018) (Fig. 1D) and the percentage of tdTomato^+^ CD16/32^+^ EMPs (35.3%) was significantly higher compared with all time points examined in the yolk sac (E8.5-E11.5, 7.07-24.5%) (Fig. 1B,C,E). Interestingly, there was an inflection point in the ratio of non-*Hoxb8* lineage (nB8L) and *Hoxb8* lineage (B8L) CD16/32^+^ EMP populations prior to establishing fetal liver hematopoiesis between E11.5 and E12.5 (Fig. 1C). By E12.5 and beyond, tdTomato^+^ CD16/32^+^ EMPs become the dominant EMP population in the fetal liver (E12.5, 72.8%; E14.5, 86.6%) (Fig. 1C), with a significant increase in number compared with B8L CD16/32^−^ and nB8L CD16/32^−^ EMP populations between E12.5 and E14.5 (Fig. S1C,D), which is likely to accommodate the production of fetal monocytes. Collectively, these data suggest that the second wave of yolk sac CD16/32^+^ EMPs, marked by the *Hoxb8* lineage, selectively expands in the fetal liver to become the main EMP population.

### Hematopoietic stem cells are derived from the *Hoxb8* lineage

**Fig. 2.**
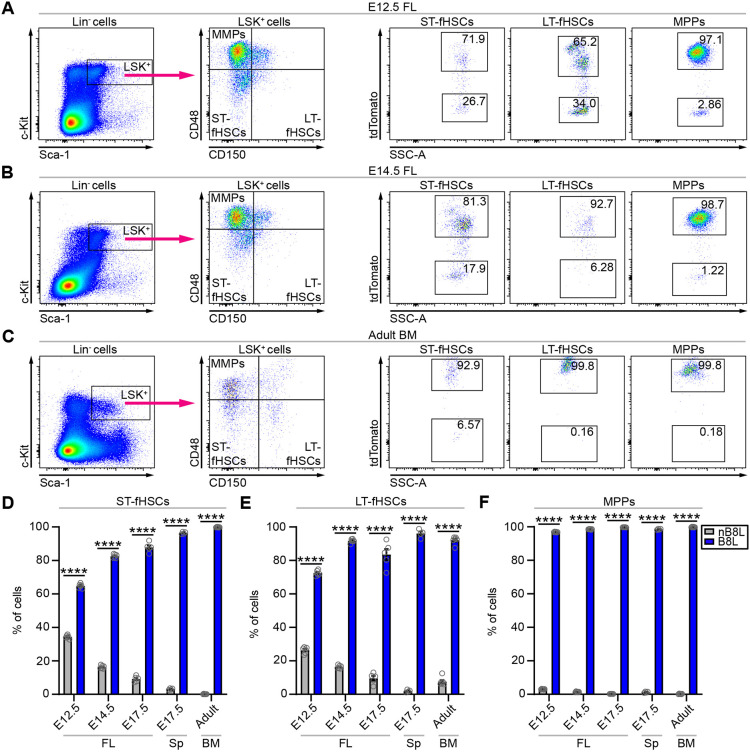
**Hematopoietic stem cells are derived from the *Hoxb8* lineage.** (A-C) Representative flow cytometry plots of Lin^−^ Sca-1^+^ Kit^hi^ (LSK^+^) cells gated for CD48 and CD150 to identify LT-fHSCs (CD150^+^ CD48^−^), ST-fHSCs (CD150^−^ CD48^−^) and MPPs (CD150^−^ CD48^+^) from fetal livers of (A) E12.5 and (B) E14.5 embryos, and (C) bone marrow of 2-month-old mice. These LSK^+^ subsets were further gated for tdTomato to identify *Hoxb8* lineage (B8L) and non-*Hoxb8* lineage (nB8L) cells. (D-F) Percentage of B8L and nB8L cells in the (D) ST-fHSC, (E) LT-fHSC and (F) MPP populations. FL, fetal liver; Sp, spleen; BM, bone marrow. *n*=6 biological replicates per group per time point; two-way ANOVA with post-hoc analysis comparing nB8L cells with B8L cells across all times points examined, *P*<0.0001. Gray circles are individual data points. Data are mean±s.e.m. ****P<0.0001. Data in all graphs are from two independent experiments per time point.

As stated in the Introduction, almost all LSK^+^ (Lineage^−^ Sca-1^+^ Kit^hi^) HSCs obtained from bone marrow of adult mice are labeled by the *Hoxb8* cell lineage marker system (De et al., 2018). As adult HSCs do not express *Hoxb8*, the lineage marker must have labeled the precursors of HSCs. Previously, we have shown that CD41^+^ and CD45^+^ Kit^+^ hematopoietic progenitors, and CD45^+^ Sca-1^+^ Kit^hi^ fHSCs can be identified in the fetal liver as early as E10.0 using the *Hoxb8*-tdTomato lineage reporter (De et al., 2018). However, the Sca-1^+^ Kit^hi^ fHSC compartment is a heterogeneous population containing subsets of short-term (ST) CD48^−^/CD150^−^ and long-term (LT) CD48^−^/CD150^+^ fHSCs, and CD48^+^ CD150^−^ multipotent progenitors (MPPs) (Pietras et al., 2015; Gudmundsson et al., 2020; Kowalczyk et al., 2015; Upadhaya et al., 2018). In the E12.5, E14.5 and E17.5 fetal liver (FL) and E17.5 spleen (Sp), the majority of ST-fHSCs (E12.5 FL: nB8L, 34.3%; B8L, 64.8%; E14.5 FL: nB8L, 16.4%; B8L, 82.6%; E17.5 FL: nB8L, 9.2%; B8L, 87.8%; E17.5 Sp: nB8L, 3.1%; B8L, 96.4%), LT-fHSCs (E12.5 FL: nB8L, 26.4%; B8L, 72.4%; E14.5 FL: nB8L, 16.4%; B8L, 91.7%; E17.5 FL: nB8L, 9.5%; B8L, 83.5%; E17.5 Sp: nB8L, 1.6%; B8L, 96.1%) and MPPs (E12.5 FL: nB8L, 3.07%; B8L, 96.9%; E14.5 FL: nB8L, 1.42%; B8L, 98.5%; E17.5 FL: nB8L, 0.21%; B8L, 99.7%; E17.5 Sp: nB8L, 1.6%; B8L, 98.5%) were tdTomato^+^ (Fig. 2A,B,D-F). In a similar fashion, the *Hoxb8*-tdTomato reporter efficiently labeled nearly all ST-HSCs (nB8L, 0.092%; B8L, 99.8%), LT-HSCs (nB8L, 7.67%; B8L, 92.1%) and MPPs (nB8L, 0.02%; B8L, 99.8%) in adult bone marrow (Fig. 2C-F), which unequivocally shows that virtually all fetal and adult HSCs and MPPs arise from *Hoxb8*-expressing precursors.

As HSCs are defined functionally by their ability to engraft and reconstitute the immune system of the recipient, we tested the reconstitution properties of *Hoxb8* lineage-derived adult LSK^+^ HSCs in immune-deficient NBSGW (*Kit^W-41J/W-41J^; Prkdc^scid/scid^; Il2rg^tm1Wjl/tm1Wjl^; Tyr^+/+^*) recipient mice. NBSGW mice allow engraftment of hematopoietic progenitors from non-autologous sources such as human without the need for pre-conditioning (i.e. radiation) (McIntosh et al., 2015). Two to three months after intra-hepatic injections of a low cell concentration of ∼2.0×10^3^ donor-derived tdTomato^+^ LSK^+^ HSCs into recipient newborn mice (Fig. S2A), engraftment of total tdTomato^+^ cells was detected in bone marrow (6.45%), spleen (6.94%), thymus (5.02%) and liver (0.28%) (Fig. S2B,C), indicating that donor cells successfully homed to their appropriate hematopoietic niches. Multi-lineage engraftment was also detected in the granulocyte (CD11b^hi^ Gr-1^hi^), monocyte (CD11b^lo^ Gr-1^int^), T cell (CD11b^−^ B220^−^) and B cell (CD11b^−^ B220^+^) compartments in spleen and bone marrow (Fig. S2D,E), suggesting that tdTomato^+^ LSK^+^ HSCs, even at a low concentration, are functionally capable of giving rise to multi-lineage hematopoietic cells.

### *Hoxb8* hematopoietic progenitors in the AGM region are selectively affected in the absence of *Myb* function

**Fig. 3.**
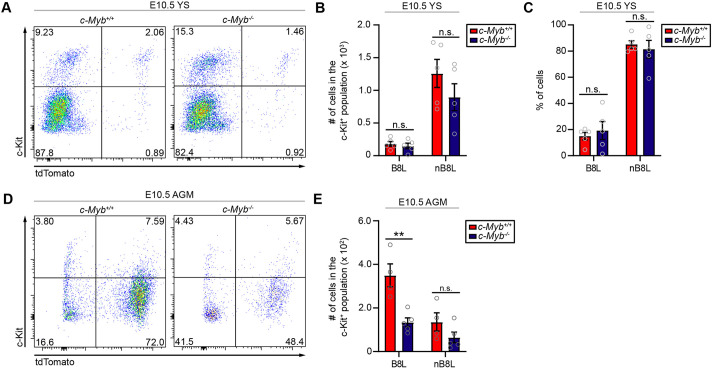
***Hoxb8* hematopoietic progenitors in the AGM are selectively affected in the absence of *Myb* function.** (A) Representative flow cytometry plots of total live Ter119^−^ Kit^+^ cells gated for tdTomato from yolk sac of E10.5 *Myb^+/+^* and *Myb^−/−^* embryos. (B) Number and (C) percentage of *Hoxb8* lineage (B8L) and non-*Hoxb8* lineage (nB8L) cells in the Kit^+^ population in the yolk sac of E10.5 *Myb^+/+^* and *Myb^−/−^* embryos. *n*=5 biological replicates per group; two-way ANOVA with post-hoc analysis comparing *Myb^+/+^* with *Myb^−/−^* in B8L [*P*=0.9909 (number), *P*=0.8153 (percentage)] and nB8L [*P*=0.2052 (number) and *P*=0.8594 (percentage)]. (D) Representative flow cytometry plots of total live Ter119^−^ Kit^+^ cells gated for tdTomato from AGM of E10.5 *Myb^+/+^* and *Myb^−/−^* embryos. (E) Number of B8L and nB8L cells in the Kit^+^ population in AGM of E10.5 *Myb^+/+^* and *Myb^−/−^* embryos. *n*=4 (*Myb^+/+^*) and *n*=5 (*Myb^−/−^*) biological replicates; two-way ANOVA with post-hoc analysis comparing *Myb^+/+^*with *Myb^−/−^* in B8L (*P*=0.0040) and nB8L (*P*=0.3300). Gray circles are individual data points. Data are mean±s.e.m. n.s. non-significant, **P<0.01. Data in all graphs are from three or four independent experiments.

Previously, we have shown that *Hoxb8* CD41^+^ and CD45^+^ Kit^+^ hematopoietic progenitors can be identified in both the yolk sac and in the AGM region. To determine in which hematopoietic tissue *Hoxb8* hematopoietic progenitors propagate and mature, we used embryos that lacked *Myb* function. Primitive hematopoiesis in the yolk sac is unaffected by the disruption of *Myb* function (Mucenski et al., 1991; Schulz et al., 2012; Sumner et al., 2000). We also did not observe a significant reduction in the number and percentage of total Ter119^−^ Kit^+^ hematopoietic progenitors in the yolk sac (Fig. 3A-C), including CD41^+^ Kit^+^ EMPs and CD45^+^ Kit^+^ progenitors (Fig. S3A,B,D,E), of either the *Hoxb8* or non-*Hoxb8* lineages in E10.5 *Myb^−/−^* embryos relative to *Myb^+/+^* embryos. These data agree with previous reports (Mucenski et al., 1991; Schulz et al., 2012; Sumner et al., 2000) that the loss of *Myb* function should not impact canonical primitive (non-*Hoxb8* lineage) hematopoietic progenitors.

We next examined the effect of disruption of *Myb* function on *Hoxb8* hematopoietic progenitors detected in the AGM region during active AGM hematopoiesis. A previous study showed that *Myb* transcripts are found predominantly in the CD45^lo/−^ Kit^+^ population in the E10.5 AGM and that, in the absence of *Myb* function in E10.5 embryos, there appears to be a reduction of the Kit-expressing population (Mizuochi et al., 2012). It has also been suggested that pre-HSCs (CD45^lo/−^ Kit^+^) are generated and mature in the P-Sp/AGM, migrate to the fetal liver (Ferkowicz et al., 2003; Rybtsov et al., 2011; Sheng et al., 2015; Taoudi et al., 2008), and acquire *Myb* function for their propagation (Mukouyama et al., 1999). We have previously shown that various Kit-expressing hematopoietic progenitor populations (>60% of CD41^+^ Kit^hi^, >80% of CD45^+^ Kit^hi^ and >90% of CD45^+^ Sca-1^+^ Kit^hi^ cells) in the E12.5 fetal liver are labeled by the *Hoxb8* cell lineage reporter (De et al., 2018). Therefore, it is reasonable to hypothesize that prior to the establishment of fetal liver hematopoiesis at E12.5, *Hoxb8* lineage Kit-expressing cells in the AGM region are dependent on *Myb* function. Indeed, Fig. 3D,E and Fig. S3C,F show that >50% of the total Ter119^−^ Kit^+^ tdTomato^+^ and Ter119^−^ CD45^lo/−^ Kit^+^ tdTomato^+^ hematopoietic progenitors (a population that includes EMPs and pre-HSCs) in the AGM region are dependent on *Myb* function. This dependence results in a ∼2-fold reduction in the number of *Hoxb8* hematopoietic progenitors in *Myb^−/−^* embryos, whereas the number of non-*Hoxb8* hematopoietic progenitors in the AGM is essentially unchanged. Notably, as yolk sac-derived EMPs migrate through the AGM region via the aorta to reach the fetal liver, the Kit^+^ population might represent migrating EMPs and emerging pre-HSCs. As a result, our data likely reflect the impact of a loss-of-function of *Myb* on both yolk sac-derived EMPs and AGM-derived pre-HSCs: hematopoietic progenitor populations that arise during definitive hematopoiesis.

### *Myb* function is required for both *Hoxb8* and non-*Hoxb8* hematopoietic progenitors during fetal liver hematopoiesis

**Fig. 4.**
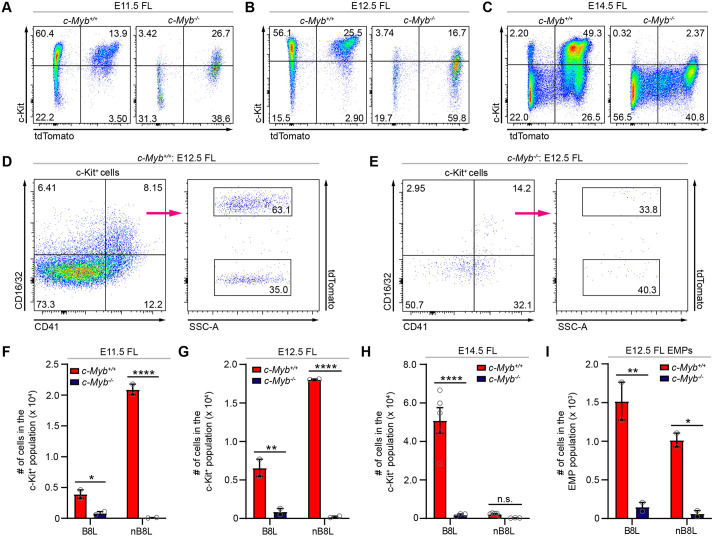
***Myb* function is required for both *Hoxb8* and non-*Hoxb8* hematopoietic progenitors during fetal liver hematopoiesis.** (A-C) Representative flow cytometry plots of Ter119^−^ Kit^+^ cells gated for tdTomato from the fetal liver (FL) of (A) E11.5, (B) E12.5 and (C) E14.5 *Myb^+/+^* and *Myb^−/−^* embryos. (D,E) Representative flow cytometry plots of CD16/32^+^ EMPs (CD41^+^ Kit^+^) gated for tdTomato from fetal livers of E12.5 (D) *Myb^+/+^* and (E) *Myb^−/−^* embryos. (F-H) Number of *Hoxb8* lineage (B8L) and non-*Hoxb8* lineage (nB8L) cells in the Kit^+^ population in fetal livers of (F) E11.5, (G) E12.5 and (H) E14.5 *Myb^+/+^* and *Myb^−/−^* embryos. E11.5: *n*=2 biological replicates per group; two-way ANOVA with post-hoc analysis comparing *Myb^+/+^* with *Myb^−/−^* in B8L (*P*=0.0351) and nB8L (*P*<0.0001). E12.5: *n*=2 biological replicates per group; two-way ANOVA with post-hoc analysis comparing *Myb^+/+^*with *Myb^−/−^* in B8L (*P*=0.0047) and nB8L (*P*<0.0001). E14.5: *n*=5 biological replicates (*Myb^+/+^*) and *n*=3 biological replicates (*Myb^−/−^*); two-way ANOVA with post-hoc analysis comparing *Myb^+/+^* with *Myb^−/−^* in B8L (*P*<0.0001) and nB8L (*P*=0.9406). (I) Number of B8L and nB8L cells in the CD16/32^+^ EMP population of the E12.5 fetal liver. *n*=2 biological replicates per group; two-way ANOVA with post-hoc analysis comparing *Myb^+/+^* with *Myb^−/−^* in B8L (*P*=0.0040) and nB8L (*P*=0.0150). Gray circles are individual data points. Data are mean±s.e.m. n.s., non-significant, *P<0.05, **P<0.01, ****P<0.0001. Data are from two or three independent experiments per time point.

We have previously reported that the *Hoxb8*-tdTomato lineage reporter labeled ∼5% of CD41^+^ Kit^hi^ hematopoietic progenitors in the yolk sac from E8.5 to E10.0, whereas >60% of CD41^+^ Kit^hi^ and 82% of CD45^+^ Kit^hi^ hematopoietic progenitors in the fetal liver were labeled from E12.5 onwards (De et al., 2018). These data suggest that the *Hoxb8*-tdTomato lineage reporter efficiently marks hematopoietic progenitor populations, including EMPs and fHSCs, during hematopoiesis in the fetal liver. As several studies have shown a loss of hematopoietic Kit^+^ progenitors in fetal livers of *Myb*^−/−^ embryos (Schulz et al., 2012; Sumner et al., 2000), we anticipated that the *Hoxb8* hematopoietic progenitor populations expressing Kit would also be strongly affected by the loss of *Myb* function. Indeed, total *Hoxb8* and non-*Hoxb8* Ter119^−^ Kit^+^ progenitors (Fig. 4A-C,F-H) and CD41^+^ Kit^hi^, CD45^lo^ Kit^hi^, and CD45^hi^ Kit^hi^ hematopoietic progenitors (Fig. S4A-C) are virtually absent in the fetal livers of E11.5, E12.5 and E14.5 *Myb^−/−^* embryos. In the fetal livers of E12.5 *Myb^−/−^* embryos, both the *Hoxb8* and non-*Hoxb8* CD16/32^+^ EMP populations are significantly reduced by the loss of *Myb* function (Fig. 4D,E,I). The complete loss of non-*Hoxb8* progenitors could represent the rapid consumption of primitive hematopoietic progenitors, as already observed in previous reports (Schulz et al., 2012). Collectively, these data suggest that EMPs from the second wave of yolk sac hematopoiesis and fHSCs from AGM hematopoiesis are compromised by the loss of function of *Myb*.

### Most fetal monocytes and tissue-resident macrophages derive from the *Hoxb8* lineage after the establishment of hematopoiesis in the fetal liver and are maintained throughout adulthood

*Hoxb8* lineage cells are detected in postnatal tissues, including peripheral blood, bone marrow and brain (Chen et al., 2010; De et al., 2018; Trankner et al., 2019). To gain a better understanding of the development of the *Hoxb8* lineage, we broadened our tracking of *Hoxb8*-tdTomato presence in the CD45^+^ pan-hematopoietic population in non-hematopoietic/lymphoid tissues (heart, liver, kidney and lung) and hematopoietic/lymphoid tissues (bone marrow, spleen, thymus and blood). CD45^+^ *Hoxb8*-tdTomato^+^ hematopoietic cells were mainly detected in hematopoietic/lymphoid tissues (BM, 75.2%; spleen, 96.9%; thymus, 98.4%) versus non-hematopoietic/lymphoid tissues (heart, 0.17%; liver, 1.35%; kidney, 7.48%; lung, 21.16%) (Fig. S5). This prompted us to survey the development of *Hoxb8* lineage committed hematopoietic cells, specifically monocytes and tissue-resident macrophages.

**Fig. 5.**
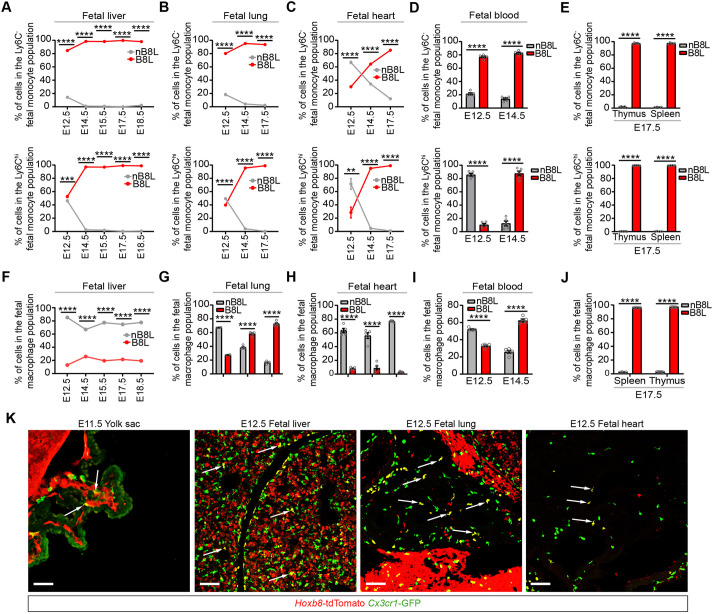
**Most fetal monocytes and tissue-resident macrophages derive from the *Hoxb8* lineage after the establishment of hematopoiesis in the fetal liver.** (A-E) Graphs showing the percentage of *Hoxb8* lineage (B8L) and non-*Hoxb8* lineage (nB8L) cells in the patrolling (CD45^+^ F4/80^−^ CD11b^+^ CD64^+^ *Cx3cr1*-GFP^+^ Ly6C^−^) and inflammatory (CD45^+^ F4/80^−^ CD11b^+^ CD64^+^ *Cx3cr1*-GFP^+^ Ly6C^hi^) fetal monocyte populations in (A) fetal liver (*n*=5-8 biological replicates per time point), (B) fetal lung (*n*=2-5 biological replicates per time point), (C) fetal heart (*n*=5 biological replicates per time point), (D) fetal blood (*n*=5 biological replicates per time point), and (E) thymus (*n*=5 biological replicates) and spleen (*n*=4 biological replicates). Two-way ANOVA with post-hoc analysis comparing nB8L cells with B8L cells across all time points examined: *P*<0.0001, except for (A) Ly6C^hi^, E12.5 fetal liver (*P*=0.0002) and (C) Ly6C^hi^, E12.5 fetal heart (*P*=0.0021). (F-J) Graphs showing the percentage of B8L and nB8L cells in the fetal macrophage (CD45^+^ F4/80^+^ CD11b^−^ CD64^+^ *Cx3cr1*-GFP^+/−^ Ly6C^−^) populations in (F) fetal liver (*n*=5-8 biological replicates per time point), (G) fetal lung (*n*=2-5 biological replicates per time point), (H) fetal heart (*n*=5 biological replicates per time point), (I) fetal blood (*n*=5 biological replicates per time point), and (J) thymus (*n*=5 biological replicates) and spleen (*n*=4 biological replicates). Two-way ANOVA with post-hoc analysis comparing nB8L cells with B8L cells across all time points examined: *P*<0.0001. Data are mean±s.e.m. **P<0.01, ***P<0.001, ****P<0.0001. Data represented in all graphs are from two to four independent experiments per time point. (K) Representative images of an E11.5 yolk sac and fetal liver, fetal lung and fetal heart from E12.5 embryos showing *Hoxb8*-tdTomato and *Cx3cr1*-GFP signals. Scale bar: 50 µm. White arrows mark tdTomato^+^ GFP^+^ cells. *n*=3 biological replicates per time point. Gray circles are individual data points.

As EMPs and fHSCs have been shown to differentiate into fetal monocytes in the fetal liver (Hoeffel et al., 2015, 2012; Hoeffel and Ginhoux, 2018; McGrath et al., 2015; Sheng et al., 2015), and the *Hoxb8*-tdTomato cell lineage reporter labels a significant fraction of these populations (De et al., 2018) (Figs 1 and 2), we looked at the development of fetal monocytes (CD45^+^ F4/80^lo^ CD11b^hi^ CD64^+^ *Cx3cr1*-GFP^+^ Ly6C^hi/−^) in embryonic tissues (fetal liver, fetal lung, fetal heart, thymus, spleen and blood) and adult hematopoietic/lymphoid tissues (bone marrow, spleen, thymus and blood) for the appearance of the *Hoxb8* lineage marker. Fetal monocytes can be separated into subsets consisting of classical inflammatory (Ly6C^hi^) and non-classical patrolling (Ly6C^−^) cells and have differential expression of Cx3cr1 (Geissmann et al., 2003; Guilliams et al., 2018; Hoeffel et al., 2015). In both the Ly6C^−^ and Ly6C^hi^ subsets, the percentage of fetal monocytes that was tdTomato^+^ (B8L) substantially increased between E12.5 and E14.5 in the fetal liver (Ly6C^−^, 84.7 to 98.4%; Ly6C^hi^, 52.6 to 97.1%), fetal lung (Ly6C^−^, 80.2 to 95.2%; Ly6C^hi^, 39.8 to 95.4%), fetal heart (Ly6C^−^, 30.1 to 64.3%; Ly6C^hi^, 28.5 to 94.9%), and circulating fetal blood (Ly6C^−^, 76.9 to 82.5%; Ly6C^hi^, 10.4 to 87.6%) (Fig. 5A-D). Notably, tdTomato^−^ (nB8L) fetal monocytes are rapidly reduced by E14.5. By E17.5, nearly all fetal monocytes are tdTomato^+^ (fetal liver: Ly6C^−^, 99.8%; Ly6C^hi^, 99.1%; fetal lung: Ly6C^−^, 93.6%; Ly6C^hi^, 98.9%; fetal heart: Ly6C^−^, 85.1%; Ly6C^hi^, 98.9%), including spleen (Ly6C^−^, 97.3%; Ly6C^hi^, 99.5%) and thymus (Ly6C^−^: 97.5%, Ly6C^hi^: 99.5%) (Fig. 5E), showing that non-*Hoxb8* lineage fetal monocytes are rapidly consumed by *Hoxb8* lineage fetal monocytes. In the adult, virtually all Ly6C^−^ and Ly6C^hi^ monocytes, and Ly6C^int^ granulocytes in each examined tissue (bone marrow, thymus, spleen) were tdTomato^+^ (Fig. S6), a result that is also detected in adult blood (Fig. S7), indicating that *Hoxb8* lineage-derived monocytes and granulocytes are maintained throughout adulthood.

As embryonic and adult spleen, thymus, blood and adult bone marrow are heavily populated with *Hoxb8* monocytes (Fig. 5, Figs S6 and S7) and *Hoxb8* HSCs (Fig. 2), we wondered whether these hematopoietic/lymphoid tissues would also contain *Hoxb8* tissue-resident macrophages. We tracked the development of *Hoxb8* tissue-resident macrophages (TRMs) in different tissues during embryonic development and during adulthood, in non-hematopoietic/lymphoid tissues (heart, liver, kidney and lung) and hematopoietic/lymphoid tissues (bone marrow, spleen and thymus). In fetal liver, the percentage of *Hoxb8* TRMs reached as high as 25.9% at E14.5 but remained significantly less than non-*Hoxb8* TRMs from E12.5 to E18.5 (Fig. 5F,K, Fig. S8). In fetal lung, whereas the percentage of nB8L TRMs decreased, the percentage of *Hoxb8* TRMs increased between E12.5 (nB8L, 62.7%; B8L, 27.5%) and E17.5 (nB8L, 16.5%; B8L, 72.7%) (Fig. 5G,K, Fig. S8). Likewise, in circulating fetal blood, an increase in percentage of *Hoxb8* TRMs was present between E12.5 (nB8L, 52.0%; B8L, 37.7%) and E14.5 (nB8L, 25.8%; B8L, 62.4%) (Fig. 5I). In fetal heart, low levels of *Hoxb8* TRMs were detected throughout development from E12.5 (nB8L, 63.3%,; B8L, 8.12%) to E17.5 (nB8L, 76.8%; B8L, 3.15%) (Fig. 5H,K, Fig. S8). By E17.5, nearly all fetal TRMs were tdTomato^+^ in thymus (nB8L, 2.72%; B8L, 96.9%) and spleen (nB8L, 2.12%; B8L, 96.1%) (Fig. 5J). Whereas other embryonic tissues had detectable levels of *Hoxb8* TRMs, such as skin (E17.5) (Fig. S8), negligible levels of *Hoxb8* TRMs were present in the yolk sac (E11.5) (Fig. 5K), midgut (E12.5), stomach (E12.5) and kidney (E14.5, E17.5) (Fig. S8).

In the adult, nearly all resident macrophage populations (CD45^+^ F4/80^+^ CD11b^lo/−^ CD64^+^ Ly6C^−^) were tdTomato^+^ (thymus: nB8L, 1.18%; B8L, 96.6%; spleen: nB8L, 0.83%; B8L, 94.8%; bone marrow: nB8L, 0.17%; B8L, 99.6%) (Fig. S9A,B), with the majority being tdTomato^+^ GFP^−^ (thymus, 84.6%; spleen, 83.0%; bone marrow, 91.1%) (Fig. S9A,C). Using the myeloid/macrophage markers Iba-1 and *Cx3cr1*-GFP, most tdTomato^+^ cells were GFP^−^ Iba1^−^ in the heart and liver, whereas lungs contained tdTomato^+^ cells that were largely Iba1^+^ GFP^+/−^ (Fig. S9D). Adult lungs were particularly interesting as ∼50% of alveolar macrophages (CD45^+^ F4/80^+^ Ly6C^−^ CD11b^−^ *Cx3cr1*-GFP^−^) (Liegeois et al., 2018) and nearly all interstitial macrophages (CD45^+^ F4/80^+^ Ly6C^−^ CD11b^+^ *Cx3cr1*-GFP^+^) (Liegeois et al., 2018) were tdTomato^+^ (Fig. S9E). The higher percentage in the lung can be explained by myeloid populations derived from the fetal liver (alveolar macrophages) and bone marrow (interstitial macrophages) (Tan and Krasnow, 2016), tissues where *Hoxb8*-tdTomato signal is widespread (De et al., 2018).

As hematopoietic/lymphoid tissues also contain other immune cells, we examined what percentage of these cells the *Hoxb8*-tdTomato lineage reporter would label. Over 93% of eosinophils, neutrophils, T cells and B cells were tdTomato^+^ (Fig. S10). Overall, these data suggest that the great majority of innate and adaptive immune cell populations in hematopoietic/lymphoid tissues are predominantly derived from *Hoxb8*-expressing precursors.

### The *Hoxb8*-tdTomato lineage reporter efficiently and selectively labels fetal macrophages derived from the second wave of yolk sac hematopoiesis

**Fig. 6.**
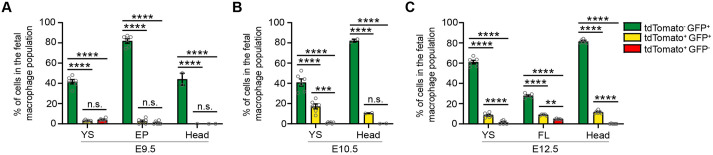
**The *Hoxb8*-tdTomato lineage reporter efficiently and selectively labels fetal macrophages derived from the second wave of yolk sac hematopoiesis.** (A-C) Flow cytometry analysis showing graphs of the percentage of tdTomato^−^ GFP^+^, tdTomato^+^ GFP^+^ and tdTomato^+^ GFP^−^ cells in the fetal macrophage populations in (A) E9.5 yolk sac (YS), embryo proper (EP) and head; (B) E10.5 YS and head; and (C) E12.5 YS, fetal liver (FL) and head. Two-way ANOVA with post-hoc analysis. (A) E9.5 YS (*n*=6 pooled biological replicates), E9.5 EP (*n*=6 pooled biological replicates), E9.5 head (*n*=2 pooled biological replicates). (B) E10.5 YS (*n*=6 pooled biological replicates), E10.5 head (*n*=2 pooled biological replicates). (C) E12.5 YS (*n*=6 biological replicates), E12.5 FL (*n*=5 biological replicates), E12.5 head (*n*=6 biological replicates). Gray circles are individual data points. Data are mean±s.e.m. n.s., non-significant. **P<0.01, ***P<0.001, ****P<0.0001. Data are from two independent experiments.

To show that the *Hoxb8*-tdTomato lineage reporter does not efficiently label primitive fetal macrophages arising during the first wave of hematopoiesis in the yolk sac (E7.5), we detected *Hoxb8* macrophages (tdTomato^+^ GFP^−^, tdTomato^+^ GFP^+^) as early as E9.5 in the yolk sac, but at significantly lower levels than non-*Hoxb8* macrophages (tdTomato^−^ GFP^+^) (tdTomato^−^ GFP^+^, 41.63%; tdTomato^+^ GFP^+^, 2.93%; tdTomato^+^ GFP^−^, 3.96%) (Fig. 6A). Similarly, we detected negligible levels in the embryo proper (tdTomato^+^ GFP^+^, 2.64%; tdTomato^+^ GFP^−^, 0.99%) and no tdTomato^+^ cells in the E9.5 head (Fig. 6A). Notably, the low level of tdTomato^+^ cells in the yolk sac could represent circulating macrophages at this time. In the E10.5 embryo, the percentage of *Hoxb8* macrophages increases in the yolk sac (tdTomato^+^ GFP^+^, 17.1%; tdTomato^+^ GFP^−^, 0.98%) and head (tdTomato^+^ GFP^+^, 10.5%; tdTomato^+^ GFP^−^, 0.22%) but remains significantly lower than non-*Hoxb8* macrophages (Fig. 6B). Similarly, *Hoxb8* macrophages remain significantly lower compared with non-*Hoxb8* macrophages in yolk sac (tdTomato^−^ GFP^+^, 61.0%; tdTomato^+^ GFP^+^, 8.49%; tdTomato^+^ GFP^−^, 1.47%), fetal liver (tdTomato^−^ GFP^+^, 27.86%; tdTomato^+^ GFP^+^, 8.82%; tdTomato^+^ GFP^−^, 4.59%) and head (tdTomato^−^ GFP^+^, 81.57%; tdTomato^+^ GFP^+^, 11.63%; tdTomato^+^ GFP^−^, 0.00%) in E12.5 embryos (Fig. 6C). These results show that a negligible percentage of primitive yolk sac-derived macrophages (first wave of hematopoiesis) arise from the *Hoxb8* lineage.

### The presence of *Hoxb8* microglia in the brain is selectively sensitive to loss of *Myb* function compared with non-*Hoxb8* microglia

**Fig. 7.**
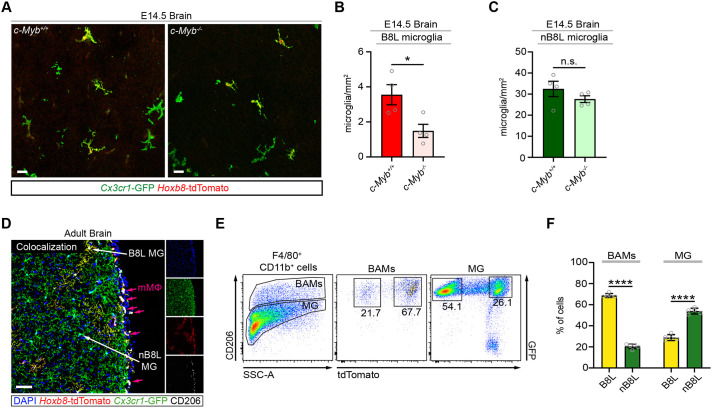
**Most border-associated macrophages and a subset of parenchymal microglia are derived from the *Hoxb8* lineage, with *Hoxb8* microglia being selectively sensitive to loss of *Myb* function.** (A) Representative images of brain sections of frontal cortex from E14.5 *Myb^+/+^* and *Myb^−/−^* embryos. Scale bars: 50 µm. (B,C) Number of (B) *Hoxb8* (B8L) microglia and (C) non-*Hoxb8* (nB8L) microglia in the brains of E14.5 *Myb^+/+^* and *Myb^−/−^* embryos. *n*=4 biological replicates per group. Unpaired *t*-test comparing *Myb^+/+^* with *Myb^−/−^* in B8L microglia (*P*=0.0238) and nB8L microglia (*P*=0.2704). (D) Representative image of a brain section of the frontal cortex from a 2-month-old *Cx3cr1^GFP/+^; Hoxb8^IRES-Cre/+^; Rosa26^CAG-LSL-tdTomato/+^* mouse stained with anti-CD206. White arrows indicate nB8L and B8L microglia (MG). Magenta arrows indicate mMΦ cells (a type of BAM) that are tdTomato^+^ GFP^+^ CD206^+^. Scale bar: 50 µm. (E) Representative flow cytometry plots examining tdTomato and GFP signals in the border-associated macrophage (BAMs: F4/80^+^ CD11b^+^ CD206^+^) and parenchymal microglia (MG: F4/80^+^ CD11b^+^ CD206^−^) populations from a brain of a 2-month-old mouse. The FACS analysis of BAMs include the mMΦ, pvMΦ and cpMΦ subsets. (F) Percentage of B8L and nB8L cells in the BAM and microglia populations. *n*=5 mice. Two-way ANOVA with post-hoc analysis comparing B8L cells with nB8L cells in both BAMs and MG populations (*P*<0.0001). Gray circles are individual data points. Data are mean±s.e.m. n.s. non-significant, *P<0.05, ****P<0.0001. Data represented in all graphs are from two independent experiments.

As the *Hoxb8*-tdTomato lineage reporter does not efficiently label primitive macrophages, including canonical non-*Hoxb8* microglia that are independent of *Myb* function during development, we asked whether non-canonical *Hoxb8* microglia could be dependent on *Myb* function. *Hoxb8* microglial progenitors, unlike canonical non-*Hoxb8* microglial progenitors, transit through the fetal liver before their migration into the developing brain at E12.5 (De et al., 2018). Because of this alternative migration route, the number of *Hoxb8* microglia present in the developing brain would be predicted to be selectively dependent on *Myb* function. The density of *Hoxb8* microglia was significantly reduced in the brains of E14.5 embryos (prior to embryonic lethality) by the absence of *Myb* function (Fig. 7A,B). In contrast, the density of non-*Hoxb8* microglia was not significantly affected in the brains of *Myb^−/−^* embryos relative to *Myb^+/+^* embryos (Fig. 7C). These results further support the unique ontogeny of *Hoxb8* microglia relative to canonical non-*Hoxb8* microglia.

### Border-associated macrophages are mainly derived from the *Hoxb8* lineage

Recent reports have identified border-associated macrophages (BAMs), which include subdural meninges macrophages (mMΦ), perivascular spaces macrophages (pvMΦ) and choroid plexus macrophages (cpMΦ), in the brain that are distinct from canonical microglia (Goldmann et al., 2016; Mrdjen et al., 2018). BAMs are first detected in the subdural meninges at E9.5 and therefore, have a prenatal origin, either arising from yolk sac or fetal liver (Goldmann et al., 2016). Using the markers CD206, *Cx3cr1*-GFP and *Hoxb8*-tdTomato, we were able to identify B8L (GFP^+^ tdTomato^+^ CD206^+^) and nB8L (GFP^+^ tdTomato^−^ CD206^+^) mMΦ BAMs and B8L (GFP^+^ tdTomato^+^ CD206^−^) and nB8L (GFP^+^ tdTomato^−^ CD206^−^) parenchymal microglia (Fig. 7D). While the percentages of B8L microglia and nB8L microglia are in agreement with prior studies (Chen et al., 2010; De et al., 2018; Nagarajan et al., 2018), the percentage of B8L BAMs (F4/80^+^ CD11b^+^ CD206^+^ GFP^+^ tdTomato^+^) (68.72%) was higher compared with nB8L BAMs (F4/80^+^ CD11b^+^ CD206^+^ GFP^+^ tdTomato^−^) (20.44%) (Fig. 7E,F), suggesting that most BAMs are of the *Hoxb8* lineage.

## DISCUSSION

The *Hoxb8* cell lineage reporter *Hoxb8^IRES-Cre^; Rosa26^CAG-LSL-tdTomato^* robustly labels hematopoietic progenitor and fHSC populations initiated during the definitive waves of yolk sac hematopoiesis at E8.5 and AGM hematopoiesis, respectively. Approximately 9% of the existing CD41^+^ Kit^+^ hematopoietic progenitor pool in the yolk sac is labeled with the *Hoxb8*-tdTomato reporter. We next observed *Hoxb8* CD41^+^ Kit^+^ hematopoietic progenitors in the AGM (E10.0-11.5), where they are expanded ∼8-fold relative to their presence in the yolk sac and represent ∼80% of the total progenitor pool (De et al., 2018) (and herein). By E12.5, *Hoxb8* CD41^+^ and CD45^+^ Kit^+^ hematopoietic progenitors are present in the fetal liver where they are further amplified (∼280-fold, again relative to their presence in the yolk sac) and comprise greater than 90% of the hematopoietic progenitor pool in the fetal liver.

Multiple aspects of the current ontological models for tissue-resident macrophages remain unresolved. For example, are tissue-resident macrophages generated from fetal monocytes (Hoeffel model) or yolk sac macrophages (Gomez-Perdiguero model)? In the current study, the yolk sac macrophages that were *Hoxb8*-tdTomato^+^ in the E9.5 yolk sac (<4%) were depleted by E10.5, whereas 98% of Ly6C^−^ and 97% of Ly6C^hi^ fetal monocytes were *Hoxb8*-tdTomato^+^ by E14.5 in the fetal liver. By comparison, our data using the *Hoxb8* lineage reporter supports the Hoeffel model. However, we were unable to completely resolve whether the contribution of fetal monocytes arises from AGM-derived fHSCs (Sheng Model) or yolk sac-derived EMPs (Hoeffel Model). We found that the great majority of LSK^+^ ST-fHSCs (83%), LT-fHSCs (91%), MPPs (98%) and E8.5 yolk sac-derived CD16/32^+^ EMPs (87%) are derived from the *Hoxb8* lineage, suggesting that Ly6C^−^ and Ly6C^hi^ fetal monocytes are derived from both hematopoietic sources. According to the Hoeffel model, E8.5 yolk sac-derived EMPs acquire *Myb* expression by E9.5, when they seed the fetal liver (Hoeffel et al., 2015; Kierdorf et al., 2013). We show that *Hoxb8* and non-*Hoxb8* CD16/32^+^ EMPs in the fetal liver are very sensitive to the presence of *Myb*, suggesting that *Myb* function is required for the great majority of the hematopoietic progenitor populations upon seeding the fetal liver. Therefore, currently, our data support both the Hoeffel and Sheng models.

We recognized that *Hoxb8* lineage CD41^+^ and CD45^+^ hematopoietic progenitors, including CD45^+^ Sca-1^+^ Kit^+^ fHSCs, can be identified in both the yolk sac, as early as E8.5, and in the AGM region, and express detectable levels of *Hoxb8* transcripts (De et al., 2018). Therefore, we hypothesized that a disruption of *Myb* function would affect the *Hoxb8* hematopoietic progenitor populations arising from definitive hematopoiesis. Whereas *Hoxb8* and non-*Hoxb8* hematopoietic progenitor cells are unaffected in the yolk sac, *Myb* function appears to specifically affect the majority of *Hoxb8* hematopoietic progenitors in the AGM region. These results provide evidence that hematopoiesis in both *Hoxb8* and non-*Hoxb8* lineages in the yolk sac is not affected by the absence of *Myb*, whereas a subset of hematopoietic progenitors derived from the *Hoxb8* lineage are selectively sensitive to *Myb* function in the AGM region during AGM hematopoiesis. In contrast, both non-*Hoxb8* and *Hoxb8* hematopoietic progenitors require *Myb* function in the fetal liver. From these experiments, we propose that the *Hoxb8*-IRES-Cre lineage mouse model firmly marks definitive hematopoiesis and efficiently labels all definitive-derived hematopoietic lineages starting in the E8.5 yolk sac.

*Hoxb8* and non-*Hoxb8* microglia have different migratory routes into the developing brain. Canonical non-*Hoxb8* microglia directly migrate from the yolk sac into the embryonic brain, starting at E9.5 as the vascular system forms (Ginhoux et al., 2010). *Hoxb8* microglia have an alternative ontogeny and passage from the yolk sac to the fetal liver before entering the developing brain starting at E12.5 (De et al., 2018). As primitive hematopoiesis is independent of *Myb* function and the production of yolk sac macrophages is still active in *Myb*-null embryos, we would anticipate that their number in the brain would not be affected by disruption of *Myb* function. On the other hand, as *Hoxb8* microglial progenitors passage through the fetal liver, their number should be significantly affected by the loss of *Myb* function. We observed a significant effect on the number of *Hoxb8* microglia, further supporting the unique ontogeny of *Hoxb8* microglia and its contribution to their unique roles in regulating homeostasis in the brain.

At E11.5, 1 day before the expansion of fetal monocytes in the fetal liver, the percentages of yolk sac-derived EMPs and AGM-derived fHSCs in the fetal liver labeled with the *Hoxb8*-tdTomato reporter are ∼41% and ∼51%, respectively, and significantly increase by E12.5 (*Hoxb8* EMPs, ∼82%; *Hoxb8* fHSCs, ∼88%). At E12.5 in the fetal liver, ∼85% of Ly6C^−^ and ∼53% of Ly6C^hi^ fetal monocytes are derived from the *Hoxb8* lineage; by E14.5, it is ∼98% and 97%, respectively. Our data, combined with the current models, suggest that yolk sac-derived *Hoxb8* EMPs and AGM-derived *Hoxb8* fHSCs generate *Hoxb8* fetal monocytes in the fetal liver, which, in turn, become *Hoxb8* tissue-resident macrophages.

Shared with our previous observations regarding adult bone marrow HSCs (Chen et al., 2010; De et al., 2018), it appears that the *Hoxb8* lineage predominantly labels hematopoietic cells and tissue-resident macrophages in tissues of active adult hematopoiesis/lymphopoiesis. These findings are congruent with our previous results that, during embryonic development, hematopoietic progenitors, AGM-derived fHSCs, yolk sac-derived EMPs and fetal monocytes are derived mainly from the *Hoxb8* lineage (De et al., 2018). Nearly all granulocytes, monocytes and lymphocytes (i.e. T cells and B cells) are of the *Hoxb8* lineage in adult blood. This makes sense because most adult innate immune blood cells are generated from LSK^+^ HSCs in the bone marrow and are labeled with the *Hoxb8*-tdTomato reporter.

Functional heterogeneity is apparent in the hematopoietic lineages. Immune cell populations contain sub-lineages with an extensive diversity of expression profiles and functions (Satpathy et al., 2012; Yona and Jung, 2010). Tissue-resident macrophages represent a diverse population of cells among tissues and within a single tissue. Their developmental origins contribute to their functional diversity and distribution in these tissues (den Haan and Kraal, 2012; Epelman et al., 2014; Hoeffel et al., 2012; Tan and Krasnow, 2016). Debate on the precise ontogeny of this diverse population of cells is ongoing (Gomez Perdiguero et al., 2015; Hoeffel et al., 2015; Sheng et al., 2015). It will be essential to determine the extent to which the developmental origin(s) and ontogeny of immune cells contribute to their diverse functions.

## MATERIALS AND METHODS

### Animals

All experiments in this study have been performed on mice. Experimental procedures were approved by the Institutional Animal Care and Use Committee at the University of Utah. *Gt(ROSA)26Sor^tm14(CAT-tdTomato)Hze^* (Ai14, 007908) and NOD.Cg-*Kit^W-41J^; Tyr^+^; Prkdc^scid^; Il2rg^tm1Wjl^/*ThomJ (NBSGW, #026622) mice were obtained from the Jackson Laboratory. *Cx3cr1^GFP^* mice (005582, Jackson Laboratory) were a kind gift from Dr Monica Vetter (Department of Neurobiology and Anatomy, University of Utah, Salt Lake City, UT, USA). *Myb^+/−^* mice (Mucenski et al., 1991) were a kind gift from Dr James Palis (Department of Pediatrics, University of Rochester Medical Center, Rochester, NY, USA). *Hoxb8^IRES-Cre^* mice are described by Chen et al. (2010).

### Flow cytometry analysis of embryonic and postnatal tissues

Embryo isolation and dissection were performed as previously described (De et al., 2018). Briefly, dissected embryos and adult tissue (2-3-months-old) were placed on ice in 10% fetal bovine serum (FBS, Atlanta Biologicals) in 1× Hanks' balanced salt solution (HBSS, Gibco). Embryonic and adult blood was collected via cardiac puncture and decapitation (embryo only). Blood samples were collected in 0.5 M EDTA-AC/10% FBS to prevent coagulation. Embryonic tissue was gently mechanically dissociated into single cells. Adult tissue was minced into small pieces. Embryonic and adult tissue was then incubated in 10% FBS/0.05% collagenase/0.025 U/ml DNaseI for 30 min at 37°C followed by a gentle mechanical dissociation into single cells. Cells were passed through a 80 µm mesh to obtain a single-cell suspension.

Anti-mouse antibodies used were as follows: TER-119 PerCP-Cy5.5 (1:50, BioLegend, 116228), B220 APC-Cy7 (1:100, BioLegend, 103224), CD11b BV510 (1:100, BioLegend, 101263), CD11b APC (1:100, BioLegend, 101212), CD115 PE-Cy7 (1:100, BioLegend, 135524), CD150 BV510 (1:100, Biolegend, 115929), CD206 Pe-Cy7 (1:100, Biolegend, 141720), CD48 PerCP-Cy5.5 (1:50, BioLegend, 103422), F4/80 PE-Cy7 (1:100, BioLegend, 123114), F4/80 PerCP-Cy5.5 (1:50, BioLegend, 123128), Kit PE-Cy7 (1:100, BioLegend, 105814), CD45 APC (1:100, BioLegend, 103112), CD16/32 APC (1:100, BioLegend, 101326), Ly6C APC-Cy7 (1:100, BioLegend, 126026), CD41 APC-eFluor 780 (1:100, eBioscience, 47-0411-80) and CD41 APC-Cy7 (1:100, BioLegend, 133928) in 10% FBS/1% BSA/1×HBSS. Cells were incubated with their respective antibody cocktail for 30 min on ice. Cells were counterstained with 3 µM DAPI in 10% FBS/1×HBSS. Flow cytometry data were obtained using the BD Bioscience FACSCanto II flow cytometry sorter or BD Bioscience FACS ARIA flow cytometry sorter. All FACS data were analyzed with FlowJo 10.0.7 (Celeza).

### Cryosectioning and immunohistochemistry

Embryonic and postnatal brain tissue were processed and sectioned as previously described (De et al., 2018). Briefly, for immunohistochemistry, brain samples were permeabilized with 0.2% Triton X-100, 1% sodium deoxycholate solution, then incubated overnight with primary antibody mixture at 4°C. The sections were incubated with secondary antibodies for 2 h at room temperature the following day. Sections were counterstained with DAPI (D1306, Molecular Probes) and mounted with ProLong Gold antifade reagent (P36934, Molecular Probes) and microscope cover glass (Fisherbrand, 22-266882). Images were acquired on the Leica TCS SP5 confocal microscope, and processed and analyzed using Imaris 7.7 (Bitplane), as described below.

Primary antibodies used were as follows: chicken anti-GFP (1:250, Aves Labs, GFP-1020), guinea pig anti-tdTomato-GP-Af430 (1:250, Frontier Institute, AB_2631185), rat anti-mouse Kit (1:250, BioLegend, 105801), rat anti-mouse CD41 (1:250, BioLegend, 133901) and rat anti-mouse CD206 Alexa Fluor 647 (1:200, Biolegend, 141712). Secondary antibodies used were: goat anti-chicken Alexa Fluor 488 (1:500, Thermo Fisher Scientific, A-11039,), goat anti-guinea pig Alexa Fluor 555 (1:500, Thermo Fisher Scientific, A-21428) and goat anti-rat Alexa Fluor 647 (1:500, Thermo Fisher Scientific, A-21247). Primary and secondary antibodies used have been tested for specificity and cross-reactivity.

### Confocal imaging parameters and Imaris image analysis

Images were acquired on a Leica TCS SP5 confocal system. For image acquisition, the tissue sections were imaged with a 10× (0.4 NA, Leica) or 20× objective (0.4 NA, Leica), and 1.5 or 2.0× digital zoom, and images were acquired at 1024×1024 resolution and 400-600 Hz scan speed, using a 5.0 µm *z*-depth through the tissue. For microglial counting, the brain sections were imaged with a 10× objective and 1.5× digital zoom, and images were acquired at 512×512 resolution and 400-600 Hz scan speed, using a 5.0 µm *z*-depth through the tissue. Images were processed using Imaris Image Analysis Software x64 (v 7.7.2, Bitplane). The ‘Spots’ function was used to count the number of microglia per unit area (mm^2^). To further identify the subsets of cells that co-label with *Cx3cr1*-GFP, the spots were filtered using ‘mean intensities’ of the fluorescence of the marker. The ‘Surface’ function was used to quantify the area of the analysis region.

### Neonatal intra-hepatic transplantations

LSK^+^ HSCs were isolated from postnatal bone marrow (P15) of *Hoxb8^IRES-Cre/+^; Rosa26^CAG-LSL-tdTomato/+^* mice and sorted by flow cytometry. Sorted *Hoxb8* LSK^+^ HSCs (∼2.0×10^3^ DAPI^−^ Lin^−^ Sca-1^+^ Kit^hi^ tdTomato^+^ cells) were transplanted into the livers of P1-P4 NBSGW mice.

### Statistical analysis

Data from all experiments were analyzed with GraphPad Prism 8 software (San Diego, CA). Unpaired *t*-test was used for direct comparison between two data groups. Standard two-way ANOVA followed by post-hoc analysis using Tukey's or Šidák's multiple comparisons test compared multiple data groups. Data are graphically reported as mean±s.e.m. (pooled and individual biological replicates). *P*<0.05 was considered significant.

## Supplementary Material

Supplementary information
