# Supplementary material for: Defining the Hoxb8 cell lineage during murine definitive hematopoiesis
Source: Development. 2022 Apr 22;149(8):dev200200. doi: 10.1242/dev.200200 (PMC9124572; doi:10.1242/dev.200200)
Supplement: Supplementary information [file develop-149-200200-s1.pdf]

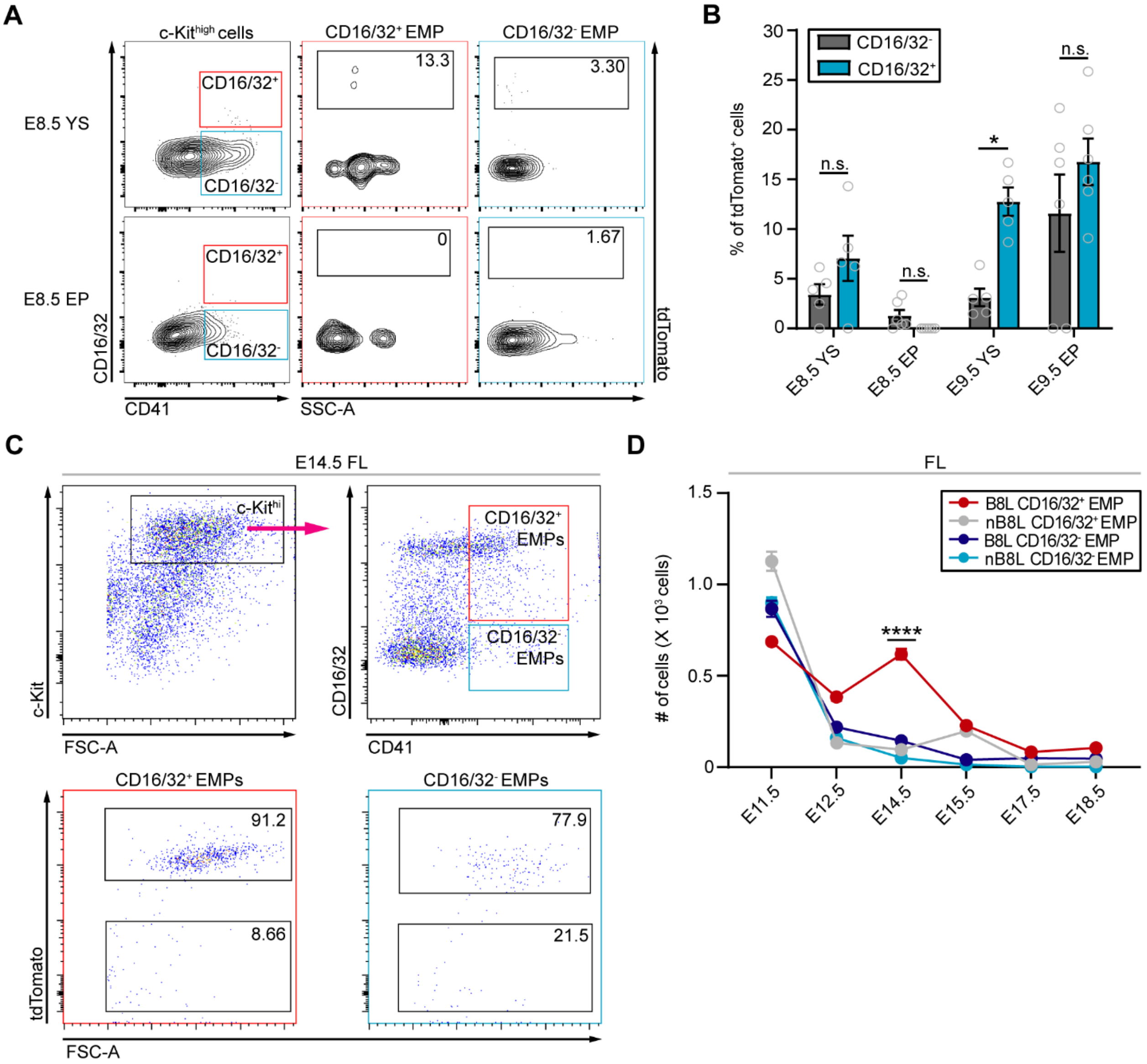

Fig. S1. Characterization of *Hoxb8* CD16/32⁻ and CD16/32⁺ EMPs during development. (**A**) Representative flow cytometry plots of CD16/32$^-$ and CD16/32$^+$ EMPs (c-Kit^{high} CD41$^+$) gated for tdTomato from E8.5 yolk sac (YS) and embryo proper (EP). (**B**) Percentage of tdTomato$^+$ cells in the CD16/32$^-$ and CD16/32$^+$ EMP populations in E8.5 and E9.5 YS and EP. 2-way ANOVA with posthoc analysis comparing CD16/32$^-$ EMPs – CD16/32$^+$ EMPs. E8.5 YS (n=5 pooled biological replicates): p=0.7001, E8.5 EP (n=6 biological replicates): p=0.9862, E9.5 YS (n=5 biological replicates): p=0.0255, E9.5 EP (n=6 biological replicates): p=0.3033. (**C**) Representative gating strategy of CD16/32$^-$ and CD16/32$^+$ EMPs (c-Kit^{hi} CD41$^+$) gated for

Development • Supplementary information

tdTomato from E14.5 fetal liver (FL). (**D**) Number of tdTomato$^+$ (B8L) and tdTomato$^-$ (nB8L) cells in the CD16/32$^-$ and CD16/32$^+$ EMP populations during fetal liver development. E11.5 FL, E12.5 FL, E14.5 FL, E18.5 FL: n=6 biological replicates. E15.5 FL, E17.5 FL: n=5 biological replicates. 2-way ANOVA with posthoc analysis, note that only the statistical analysis comparing B8L CD16/32$^+$ EMPs to all other EMP populations at E14.5 is shown: $p<0.0001$. Data are represented as mean±sem. n.s. non-significant, *$p<0.05$, ****$p<0.0001$. Data represented in all graphs are from two to three independent experiments per time point.

Development • Supplementary information

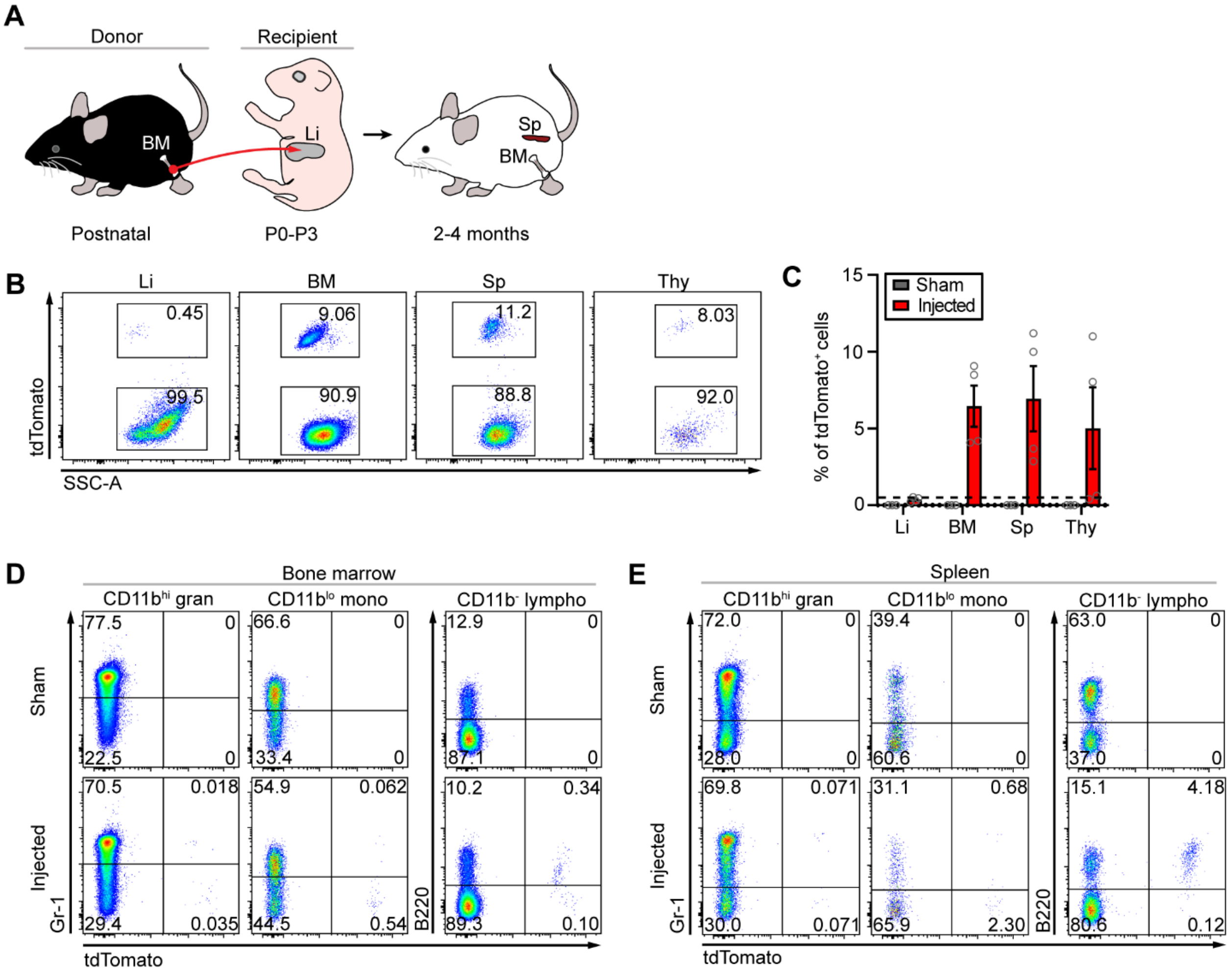

Fig. S2. *Hoxb8* HSCs have multilineage hematopoietic capacity. (**A**) Illustration showing the intra-hepatic injection of postnatal bone marrow (BM)-derived HSCs into a liver of a neonatal (P0-P3) recipient mouse that lacks a functional immune system (NBSGW). Liver (Li), Spleen (Sp), Thymus (Thy), and BM were harvested 2-4 months following transplantation. (**B**) Representative flow cytometry plots showing the total percentage of donor-derived tdTomato$^+$ and endogenous tdTomato$^-$ live cells in the examined tissues. (**C**) Percentage of donor-derived tdTomato$^+$ cells in examined tissues. n=3 biological replicates (sham injected) and n=4 biological replicates (cell injected). Gray circles represent individual data points. Data are represented as mean±sem. The dotted line marks the minimum threshold for transplantation efficiency (>0.1%). Note that >5% of donor-derived tdTomato$^+$ cells were detected in

Development • Supplementary information

hematopoietic/lymphoid tissues in adult mice. (**D-E**) Representative flow cytometry plots showing $CD11b^{hi}$ granulocytes (gran), $CD11b^{lo}$ monocytes (mono), and $CD11b^{-}$ lymphocytes (lympho) gated for tdTomato and Gr-1 in (**D**) bone marrow and (**E**) spleens of sham-injected and cell-injected recipient mice. Data represented in all graphs are from two independent experiments.

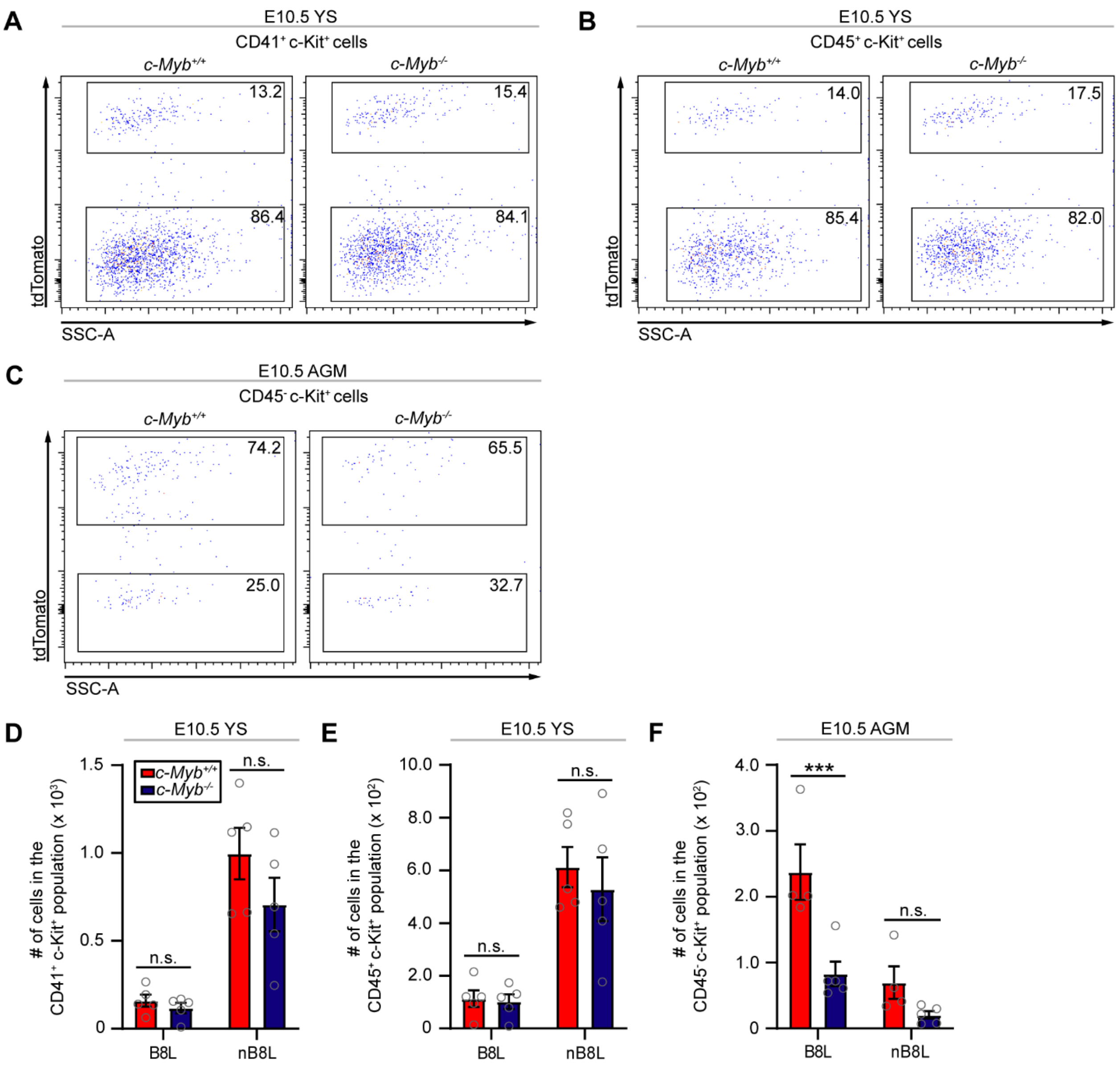

Fig. S3. AGM-derived *Hoxb8* hematopoietic progenitors require *c-Myb* function. (**A-C**) Representative flow cytometry plots showing (**A**) CD41^{+} c-Kit^{+} and (**B**) CD45^{+} c-Kit^{+} cells in yolk sac (YS) and (**C**) CD45^{-} c-Kit^{+} cells in AGM gated for tdTomato in E10.5 *c-Myb*$^{+/+}$ and *c-Myb*$^{-/-}$ embryos. (**D-F**) Number of *Hoxb8* lineage (B8L) and non-*Hoxb8* lineage (nB8L) cells in the (**D**) CD41^{+} c-Kit^{+} population in yolk sac (n=5 biological replicates per group, 2-way ANOVA with posthoc analysis comparing *c-Myb*$^{+/+}$ – *c-Myb*$^{-/-}$ in B8L: *p*=0.9761 and nB8L: *p*=0.3868), (**E**) CD45^{+} c-Kit^{+} populations in yolk sac (n=5 biological replicates per group, 2-way ANOVA with posthoc analysis comparing *c-Myb*$^{+/+}$ – *c-Myb*$^{-/-}$ in B8L: *p*=0.9943 and nB8L: *p*=0.7389), and (**F**) CD45^{-} c-Kit^{+} population in AGM (2-way ANOVA with posthoc analysis

Development • Supplementary information

comparing *c-Myb*$^{+/+}$ (n=4 biological replicates) – *c-Myb*$^{-/-}$ (n=5 biological replicates) in B8L: *p*=0.0176 and nB8L: *p*=0.6739) from E10.5 *c-Myb*$^{+/+}$ and *c-Myb*$^{-/-}$ embryos. Gray circles represent individual data points. Data are represented as mean±sem. n.s. non-significant, ****p*<0.001. Data represented in all graphs are from three to four independent experiments.

Development • Supplementary information

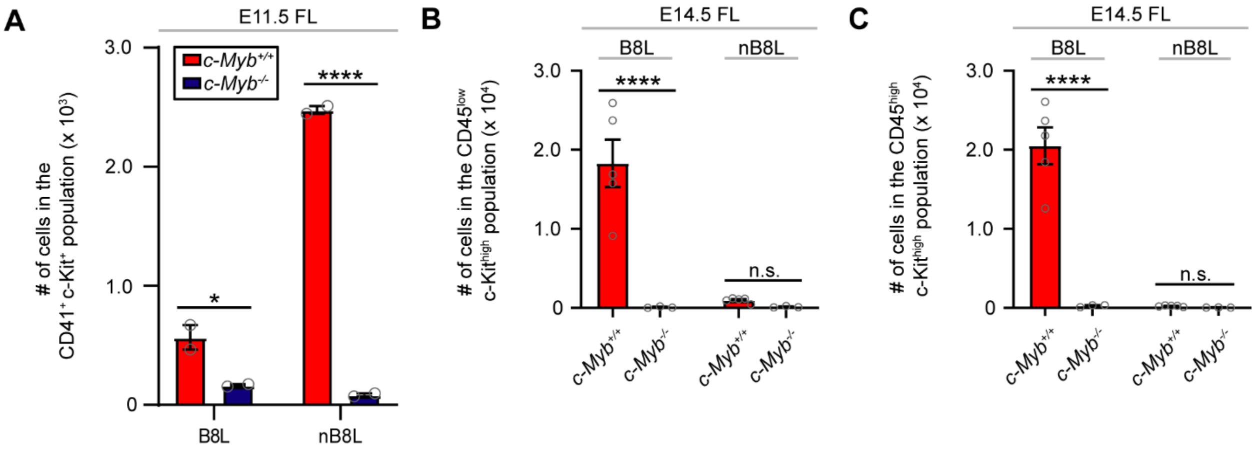

Fig. S4. The absence of *c-Myb* function in fetal liver disrupts the propagation of maturing hematopoietic progenitors. (**A**) Number of *Hoxb8* lineage (B8L) and non-*Hoxb8* lineage (nB8L) cells in the $CD41^+$ $c\text{-}Kit^+$ population in fetal liver (FL) of E11.5 $c\text{-}Myb^{+/+}$ and $c\text{-}Myb^{-/-}$ embryos. n=2 biological replicates per group; 2-way ANOVA with posthoc analysis comparing $c\text{-}Myb^{+/+}$ – $c\text{-}Myb^{-/-}$ in B8L: p=0.0124 and nB8L: p<0.0001. (**B**) Number of B8L and nB8L cells in the $CD45^{lo}$ $c\text{-}Kit^{high}$ population in fetal liver of E14.5 $c\text{-}Myb^{+/+}$ and $c\text{-}Myb^{-/-}$ embryos. 2-way ANOVA with posthoc analysis comparing $c\text{-}Myb^{+/+}$ (n=5 biological replicates) – $c\text{-}Myb^{-/-}$ (n=3 biological replicates) in B8L: p<0.0001 and nB8L: p=0.9481. (**C**) Number of B8L and nB8L cells in the $CD45^{high}$ $c\text{-}Kit^{high}$ population in fetal liver of E14.5 $c\text{-}Myb^{+/+}$ and $c\text{-}Myb^{-/-}$ embryos. 2-way ANOVA with posthoc analysis comparing $c\text{-}Myb^{+/+}$ (n=5 biological replicates) – $c\text{-}Myb^{-/-}$ (n=3 biological replicates) in B8L: p<0.0001 and nB8L: p=0.9963. Gray circles are individual data points. Data are represented as mean±sem. n.s. non-significant, *p<0.05, ****p<0.0001. Data represented in all graphs are from two to three independent experiments per time point.

Development • Supplementary information

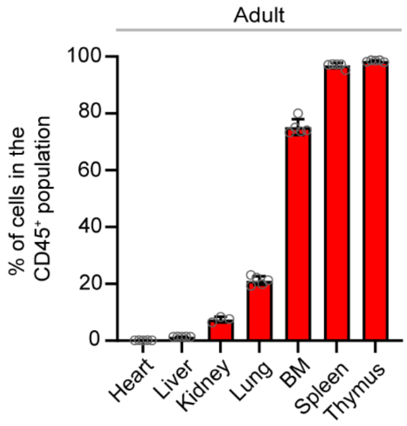

Fig. S5. *Hoxb8* CD45$^+$ hematopoietic cells are concentrated in adult hematopoietic/lymphoid tissues. Graph showing the percentage of live Ter119$^-$ CD45$^+$ *Hoxb8*-tdTomato$^+$ hematopoietic cells in examined tissues of 2-3-month-old mice. n=5 biological replicates (Heart), n=5 biological replicates (Liver), n=3 biological replicates (Kidney), n=5 biological replicates (Lung), n=5 biological replicates (Bone marrow: BM), n=5 biological replicates (Spleen), n=5 biological replicates (Thymus). Data are represented as mean±sem. Data represented in all graphs are from two to four independent experiments per time point.

Development • Supplementary information

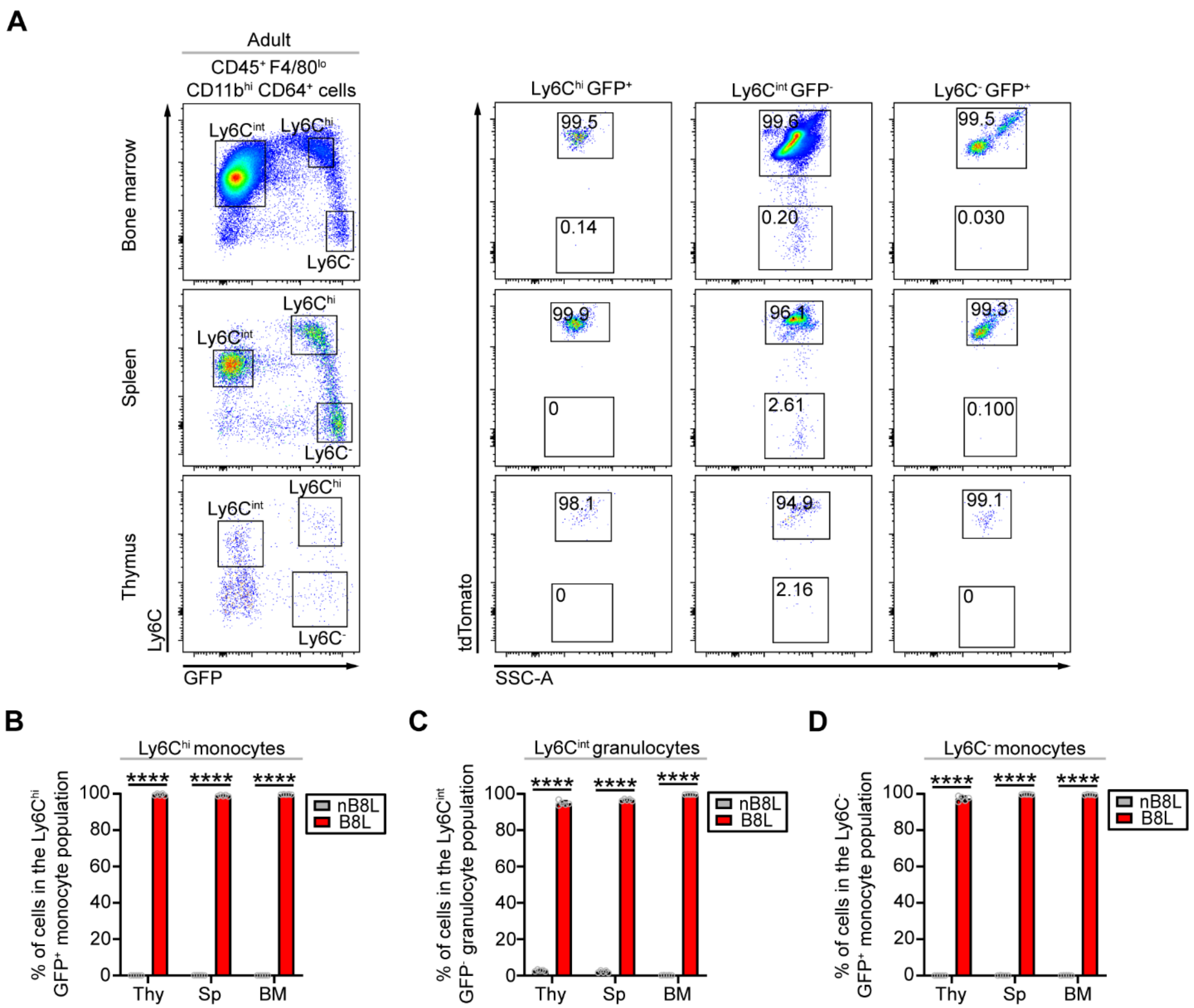

Fig. S6. Inflammatory and patrolling monocytes, including granulocytes, in adult tissues of hematopoiesis/lymphopoiesis, are descendants of *Hoxb8*-expressing precursors. (**A**) Representative flow cytometry plots examining tdTomato^{+} cells in the inflammatory (CD45^{+} F4/80^{-} CD11b^{+} CD64^{+} *Cx3cr1*-GFP^{+} Ly6C^{hi}) and patrolling (CD45^{+} F4/80^{-} CD11b^{+} CD64^{+} *Cx3cr1*-GFP^{+} Ly6C^{-}) monocyte populations, and the granulocyte population (CD45^{+} F4/80^{-} CD11b^{+} CD64^{+} *Cx3cr1*-GFP^{-} Ly6C^{int}) from bone marrow, spleen, and thymus of 2-3-month-old mice. (**B-D**) Percentage of *Hoxb8* lineage (B8L) and non-*Hoxb8* lineage (nB8L) cells in the (**B**) Ly6C^{hi} inflammatory monocyte, (**C**) Ly6C^{int} granulocyte, and (**D**) Ly6C^{-} patrolling monocyte populations in thymus (Thy), spleen (Sp), and bone marrow (BM). n=5 biological replicates. 2-

Development • Supplementary information

way ANOVA with posthoc analysis comparing nB8L cells – B8L cells in each tissue examined: $p<0.0001$. Gray circles are individual data points. Data are represented as mean±sem. ****$p<0.0001$. Data represented in all graphs are from two to four independent experiments per time point.

Development • Supplementary information

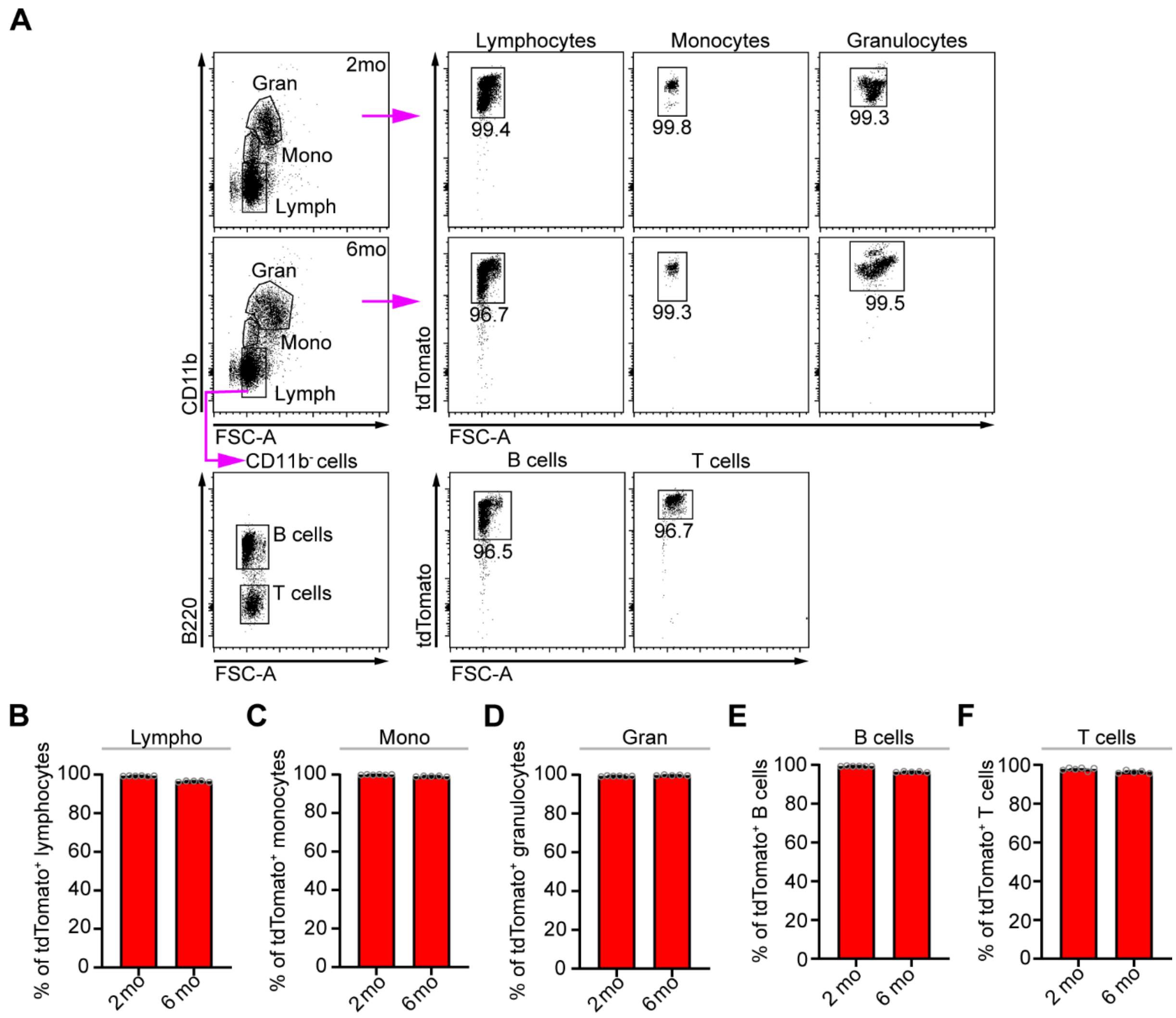

Fig. S7. All blood immune cells are derived from the *Hoxb8* lineage. (**A**) Representative flow cytometry plots showing the frequencies of white blood cells ($CD11b^-$ lymphocytes: Lymph, $CD11b^{lo}$ monocytes: Mono, $CD11b^{hi}$ granulocytes: Gran) from 2-month and 6-month-old adult mice. Lymphocytes were further gated for B220 to examine B cells ($CD11b^-$ $B220^+$) and T cells ($CD11b^-$ $B220^-$) followed by gating for tdTomato. (**B-D**) Percentage of $tdTomato^+$ (**B**) lymphocytes, (**C**) monocytes, and (**D**) granulocytes in adult blood. n=6 biological replicates (2-months-old) and n=5 biological replicates (6-months-old). (**E-F**) Percentage of $tdTomato^+$ cells in the (**E**) B cell and (**F**) T cell populations of adult mice. n=6 biological replicates (2-months-old) and n=5 biological replicates (6-months-old). Data are represented as mean±sem. Data represented in all graphs are from two to four independent experiments per time point.

Development • Supplementary information

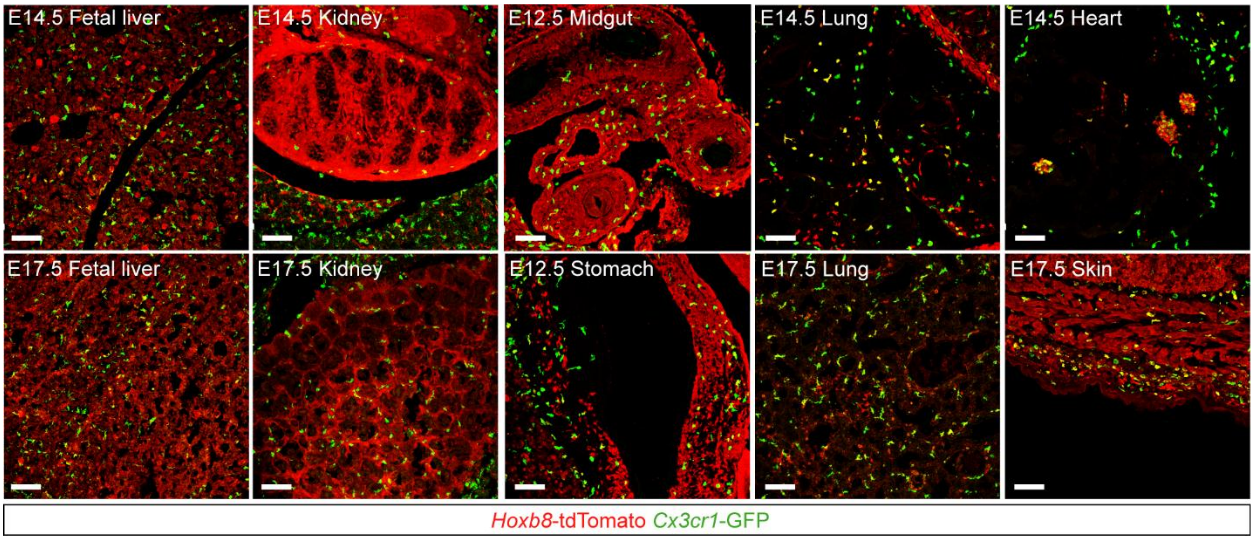

Fig. S8. Development of *Hoxb8* lineage and non-*Hoxb8* lineage fetal macrophages.

Micrographs of sections from embryonic tissues showing *Cx3cr1*-GFP and *Hoxb8*-tdTomato signals. Scale bar: 50µm. n=3 biological replicates per time point.

Development • Supplementary information

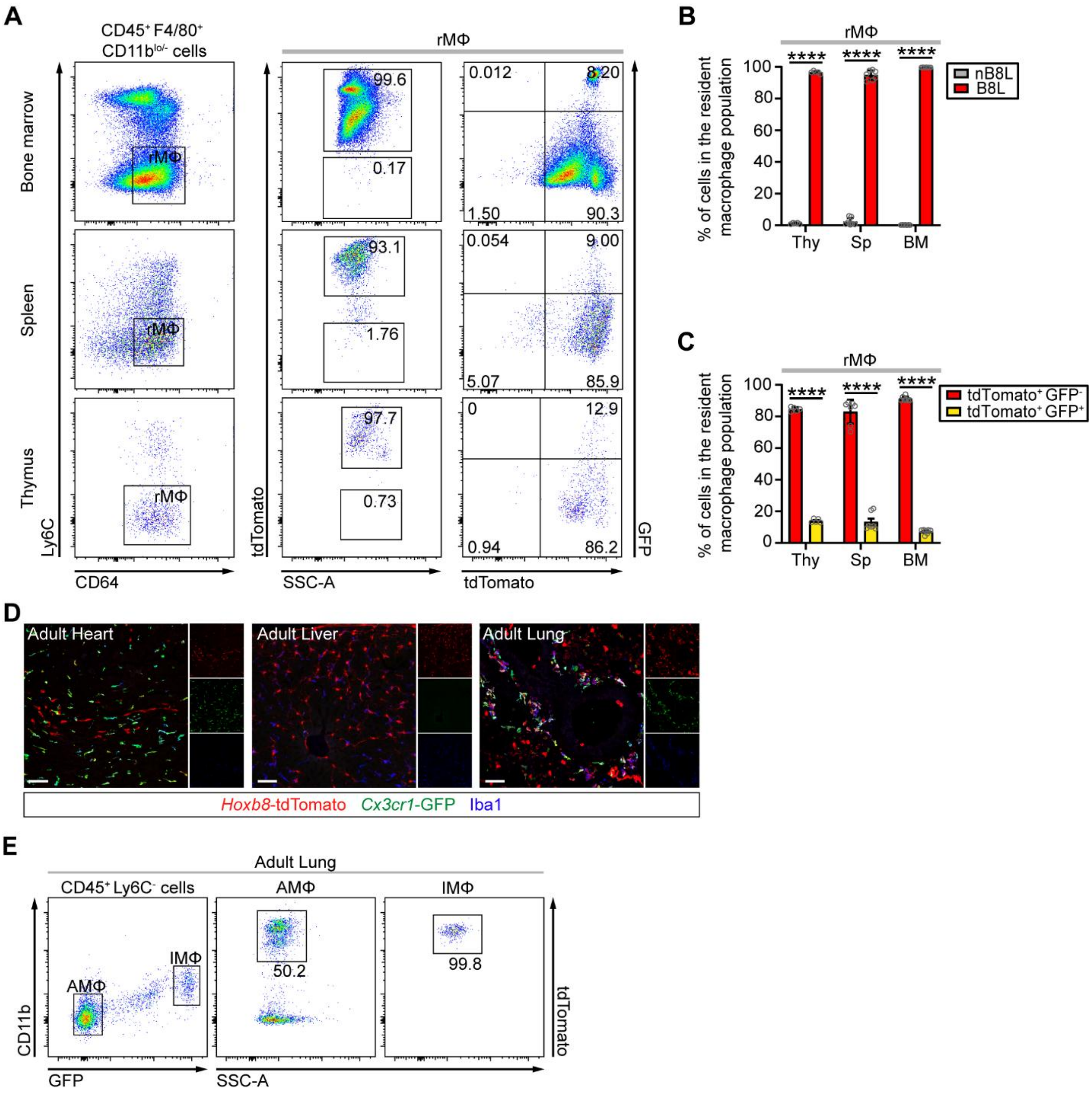

Fig. S9. *Hoxb8* tissue-resident macrophages are largely found in tissues undergoing active hematopoiesis/lymphopoiesis. (**A**) Representative flow cytometry plots examining total tdTomato (SSC-A vs. tdTomato) or tdTomato and GFP (tdTomato vs. GFP) signals in the resident (rMΦ: $CD45^+$ $F4/80^+$ $CD11b^{lo/-}$ $CD64^+$ $Ly6C^-$) macrophage populations from bone marrow, spleen, and thymus of 2-3-month-old adult mice. (**B-C**) Percentage of (**B**) *Hoxb8* lineage (B8L) and non-*Hoxb8* lineage (nB8L) cells and (**C**) $tdTomato^+$ GFP^- and $tdTomato^+$ GFP^+ cells in the rMΦ population of 2-3-month-old mice. Thymus (n=5 biological replicates),

Development • Supplementary information

spleen and bone marrow (n=7 biological replicates). (**B**) 2-way ANOVA with posthoc analysis comparing nB8L cells – B8L cells in each tissue examined, $p<0.0001$. (**C**) 2-way ANOVA with posthoc analysis comparing tdTomato^{+} GFP^{-} cells – tdTomato^{+} GFP^{+} cells in the rMΦ population in each tissue examined: $p<0.0001$. (**D**) Micrographs of sections from non-hematopoietic tissues (heart, liver, lung) of 2-3-month-old mice showing *Cx3cr1*-GFP, *Hoxb8*-tdTomato, and Iba1 signals. Scale bar: 50µm. n=3 biological replicates. (**E**) Representative flow cytometry plots showing alveolar (AMΦ: CD45^{+} Ly6C^{-} CD11b^{-} *Cx3cr1*-GFP^{-}) and interstitial (IMΦ: CD45^{+} Ly6C^{-} CD11b^{+} *Cx3cr1*-GFP^{+}) macrophage populations gated for tdTomato in lungs of 2-3-month-old mice. Gray circles are individual data points. Data are represented as mean±sem. ****$p<0.0001$. Data represented in all graphs are from two to four independent experiments per time point.

Development • Supplementary information

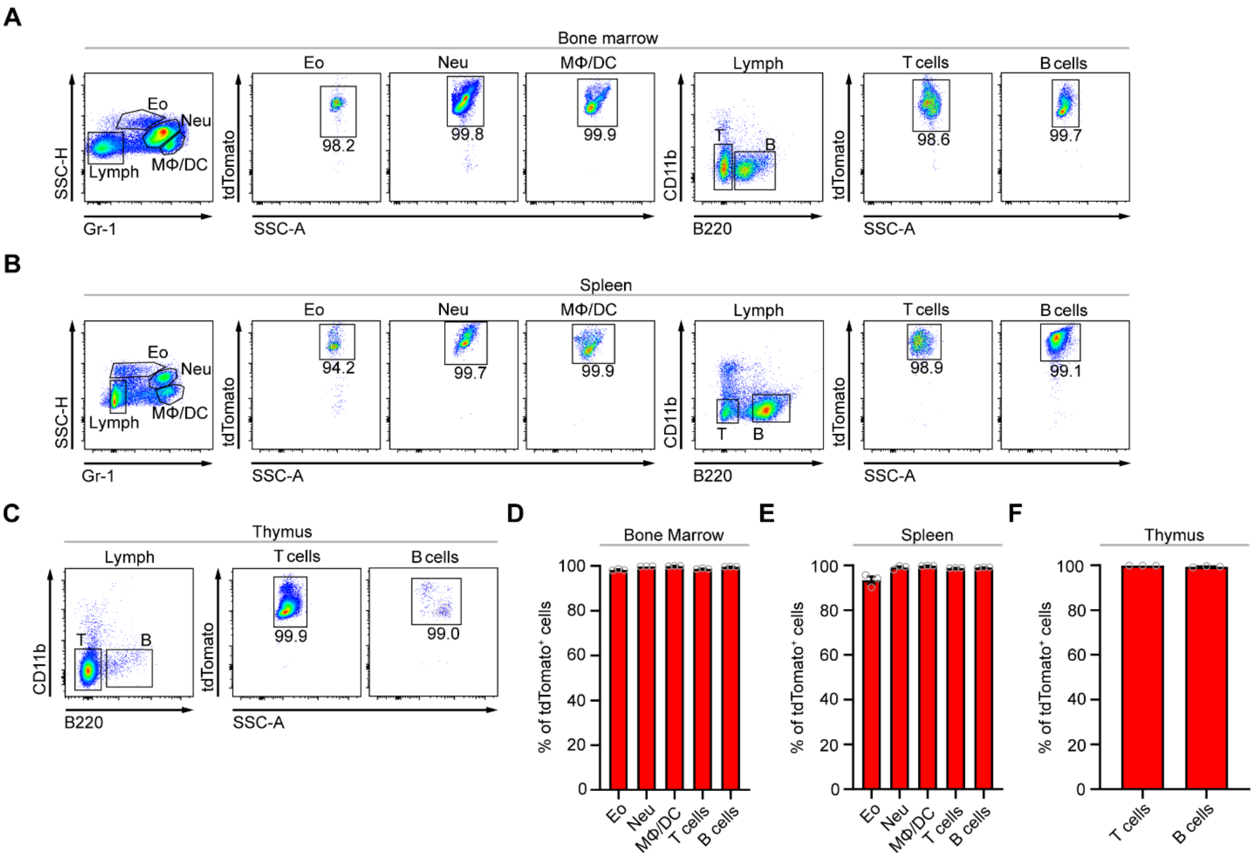

Fig. S10. Innate and adaptive immune cells in tissues of active hematopoiesis/lymphopoiesis are exclusively descendants of the *Hoxb8*-expressing precursors. (**A-C**) Representative flow cytometry plots examining tdTomato^{+} cells in the eosinophil (Eo) (Gr-1$^{low/-}$), neutrophil (Neu) (Gr-1^{med}), macrophage/dendritic cell (MΦ/DC) (Gr-1^{hi}), and lymphocyte (Lymph) (Gr-1^{-}) populations isolated from (**A**) bone marrow, (**B**) spleen, and (**C**) thymus of 2-3-month-old mice. Gr-1^{-} lymphocytes were further examined for tdTomato detection in T cells (T) (CD11b^{-} B220^{-}) and B cells (B) (CD11b^{-} B220^{+}). (**D-F**) Percentage of tdTomato^{+} cells in each immune cell population examined in (**D**) bone marrow, (**E**) spleen, and (**F**) thymus. n=3 biological replicates. Gray circles represent individual data points. Data are represented as mean±sem. Data represented in all graphs are from two independent experiments.

Development • Supplementary information
